# *MICB*^G406A^ polymorphism reduces risk of mechanical ventilation and death during viral acute lung injury

**DOI:** 10.1172/jci.insight.191951

**Published:** 2025-07-03

**Authors:** Harry Pickering, Narges Alipanah-Lechner, Ernie Chen, Dylan Duchen, Holden T. Maecker, Seunghee Kim-Schulze, Ruth R. Montgomery, Chris Cotsapas, Hanno Steen, Florian Krammer, Charles R. Langelier, Ofer Levy, Lindsey R. Baden, Esther Melamed, Lauren I.R. Ehrlich, Grace A. McComsey, Rafick P. Sekaly, Charles B. Cairns, Elias K. Haddad, Albert C. Shaw, David A. Hafler, David B. Corry, Farrah Kheradmand, Mark A. Atkinson, Scott C. Brakenridge, Nelson I. Agudelo Higuita, Jordan P. Metcalf, Catherine L. Hough, William B. Messer, Bali Pulendran, Kari C. Nadeau, Mark M. Davis, Ana Fernandez Sesma, Viviana Simon, Monica Kraft, Chris Bime, David J. Erle, Joanna Schaenmann, Al Ozonoff, Bjoern Peters, Steven H. Kleinstein, Alison D. Augustine, Joann Diray-Arce, Patrice M. Becker, Nadine Rouphael, Matthew C. Altman, Steve Bosinger, Walter Eckalbar, IMPACC Network, Carolyn S. Calfee, Oscar A. Aguilar, Elaine F. Reed, John R. Greenland, Daniel R. Calabrese

**Affiliations:** 1 Pathology and Laboratory Medicine, UCLA, Los Angeles, California, USA.; 2 Department of Medicine, UCSF, San Francisco, California, USA.; 3 Yale School of Medicine, New Haven, Connecticut, USA.; 4 Stanford University School of Medicine, Palo Alto, California, USA.; 5 Icahn School of Medicine at Mount Sinai, New York, New York, USA.; 6 Precision Vaccines Program, Boston Children’s Hospital, and; 7 Brigham and Women’s Hospital, Harvard Medical School, Boston, Massachusetts, USA.; 8 The University of Texas at Austin, Austin, Texas, USA.; 9 Case Western Reserve University and University Hospitals of Cleveland, Cleveland, Ohio, USA.; 10 Drexel University, Tower Health Hospital, Philadelphia, Pennsylvania, USA.; 11 Baylor College of Medicine and the Center for Translational Research on Inflammatory Diseases, Houston, Texas, USA.; 12 University of Florida, Gainesville, Florida, USA.; 13 Oklahoma University Health Sciences Center, Oklahoma City, Oklahoma, USA.; 14 Oregon Health & Science University, Portland, Oregon, USA.; 15 University of Arizona, Tucson, Arizona, USA.; 16 La Jolla Institute for Immunology, La Jolla, California, USA.; 17 National Institute of Allergy and Infectious Diseases, National Institute of Health, Bethesda, Maryland, USA.; 18 Emory School of Medicine, Atlanta, Georgia, USA.; 19 Benaroya Research Institute, University of Washington, Seattle, Washington, USA.; 20 Emory School of Medicine, Atlanta, Georgia, USA.; 21 The Immunophenotyping Assessment in a COVID-19 Cohort (IMPACC) Network is detailed in Supplemental Acknowledgments.; 22 Department of Microbiology & Immunology and; 23 Parker Institute for Cancer Immunotherapy, UCSF, San Francisco, California, USA.; 24 Medical Service, San Francisco VA Health Care System, San Francisco, California, USA.

**Keywords:** Immunology, Pulmonology, COVID-19, Innate immunity, NK cells

## Abstract

MHC class I polypeptide-related sequence B (MICB) is a ligand for NKG2D. We have shown NK cells are central to lung transplant acute lung injury (ALI) via NKG2D activation, and increased MICB in bronchoalveolar lavage predicts ALI severity. Separately, we found a MICB polymorphism (*MICB*^G406A^) is associated with decreased ALI risk. We hypothesized this polymorphism would protect against severe SARS-CoV-2 respiratory disease. We analyzed 1,036 patients hospitalized with SARS-CoV-2 infection from IMPACC. Associations between *MICB*^G406A^ and outcomes were determined by linear regression or Cox proportional hazards models. We also measured immune profiles of peripheral blood and the upper and lower airway. We identified 560 major allele homozygous patients, and 426 and 50 with 1 or 2 copies of the variant allele, respectively. *MICB*^G406A^ conferred reduced odds of severe COVID-19. MICB^G406A^ homozygous participants demonstrated 34% reduced cumulative odds for mechanical ventilation or death and 43% reduced risk for mortality. Patients with MICB^G406A^ variant alleles had reduced soluble inflammatory mediators and differential regulation of multiple immune pathways. These findings demonstrate a potentially novel association between increasing *MICB*^G406A^ variant allele copies and reduced COVID-19 severity, independent of SARS-CoV-2 viral burden and humoral immunity, suggesting the NKG2D-ligand pathway as an intervention target.

## Abstract 2

MICB, a ligand for the NK cell NKG2D receptor, has previously been associated with lung allograft injury. We found that a polymorphism in this gene was associated with COVID outcomes.

## Introduction

Acute lung injury (ALI) is a pathologic process that occurs from a host of risk factors, but it is strongly associated with pneumonia from sterile, bacterial, or viral etiologies (1). Consequently, SARS-CoV-2 is a significant driver of ALI (2). Acute respiratory distress syndrome (ARDS) is the clinical syndrome of severe ALI described as impaired oxygenation and bilateral opacifications on chest imaging (3). There are over 200,000 US cases of ARDS annually (2) that account for 10% of intensive care unit (ICU) admissions (4) with up to 40% attributable mortality (5). The mainstay of treatment for ARDS remains supportive care (6); thus, there is an interest in defining pathologic processes underpinning ARDS for better therapeutic targeting.

ALI results from pulmonary endothelial and epithelial injury and dysfunction, leading to inflammatory mediator release (7–9) and innate immune cell activation (10, 11). NK cells are innate lymphocytes (12) that influence lung health through surveillance of missing self (lack of MHC class I) (13, 14), pathogenic (15), or “stressed” cells (16). Their actions are determined by the integration of inhibitory and activating signals through somatically encoded surface receptor ligation (17). We have recently described that NK cells mediate ischemia-reperfusion injury (IRI) after lung transplantation (18–20), which shares basic pathophysiology with ARDS. We previously identified that NK cell activation in IRI occurs through the NKG2D receptor recognition of proteins expressed on epithelial, endothelial, and immune cells under stress from infection, hypoxia, DNA damage, or a range of other stimuli. NKG2D receptor engagement with 1 of the 8 human stress ligands leads to direct target cell cytotoxicity and NK cell release of inflammatory cytokines. One of the human NKG2D stress ligands, MHC class I polypeptide-related sequence B (MICB), is highly expressed throughout the lung, and increased soluble MICB is associated with ALI (19).

NK cells have been implicated in SARS-CoV-2 pathogenesis, with several groups observing a phenotype with increased activation markers and features of memory (21, 22) in severe SARS-CoV-2 infection. However, dysregulation in the circulating NK cell compartment has also been linked to ALI after COVID-19 (23). Genetic polymorphisms in key NK cell receptors have been linked to pulmonary disease pathogenesis. Notably, mutations in perforins, released by NK cells to kill target cells, have been associated with fatal influenza (24). A polymorphism in the Fc activating receptor CD16a conferring increased affinity for IgG, enhancing NK cell antibody-dependent functions, has been observed in severe and fatal cases of SARS-CoV-2 infection (25). Finally, differences in Human Leukocyte Antigen (HLA) and their cognate NK cell receptors Killer Immunoglobulin-like Receptor (KIR) have also been implicated in SARS-CoV-2 pathology (26). Our group has previously shown that an intronic, missense single nucleotide (NT) polymorphism (SNP) in the lung (transplant donor) *MICB* stress ligand gene conferred reduced odds of severe ALI after transplant (20). We also showed that this *MICB^G406A^* SNP (rs1051788) was associated with reduced surface and bronchoalveolar lavage (BAL) MICB protein, and it led to reduced and less cytotoxic NK cells in the BAL. However, the role of *MICB^G406A^*, and the NKG2D pathway, in risk for SARS-CoV-2 clinical sequelae remains unknown.

There is evidence that hyperresponsive NK cells are enriched in the lung, setting the stage for their role in more severe forms of ARDS (27). There have been many attempts to classify ARDS by physiologic traits associated with mortality risk (28). ARDS severity grades (29), dead space (30, 31), and driving pressure (32) are 3 key examples. However, these ARDS traits occur late and are prone to misclassification. Using latent class analysis (LCA), a statistical approach used for unsupervised grouping, 2 subphenotypes of ARDS have been recognized: hypoinflammatory and hyperinflammatory (33–35). Hyperinflammatory ARDS is characterized by increased vasopressor use, reduced ventilator-free days, and increased mortality relative to the hypoinflammatory subtype (35). In hyperinflammatory ARDS, NK cells and their receptors have been identified in the lung (5).

Here, we hypothesized that the *MICB^G406A^* polymorphism would be associated with less severe COVID-19 and improved clinical outcomes. We studied IMPACC of patients hospitalized with SARS-CoV-2 infection to investigate the effect of the *MICB^G406A^* polymorphism on severity of COVID-19. Additionally, peripheral blood and airway multi-omic profiling was used to explore how the *MICB^G406A^* polymorphism influences immune response to SARS-CoV-2 infection.

## Results

### MICB^G406A^ genotype and associated patient demographics of IMPACC.

**Figure 1:**
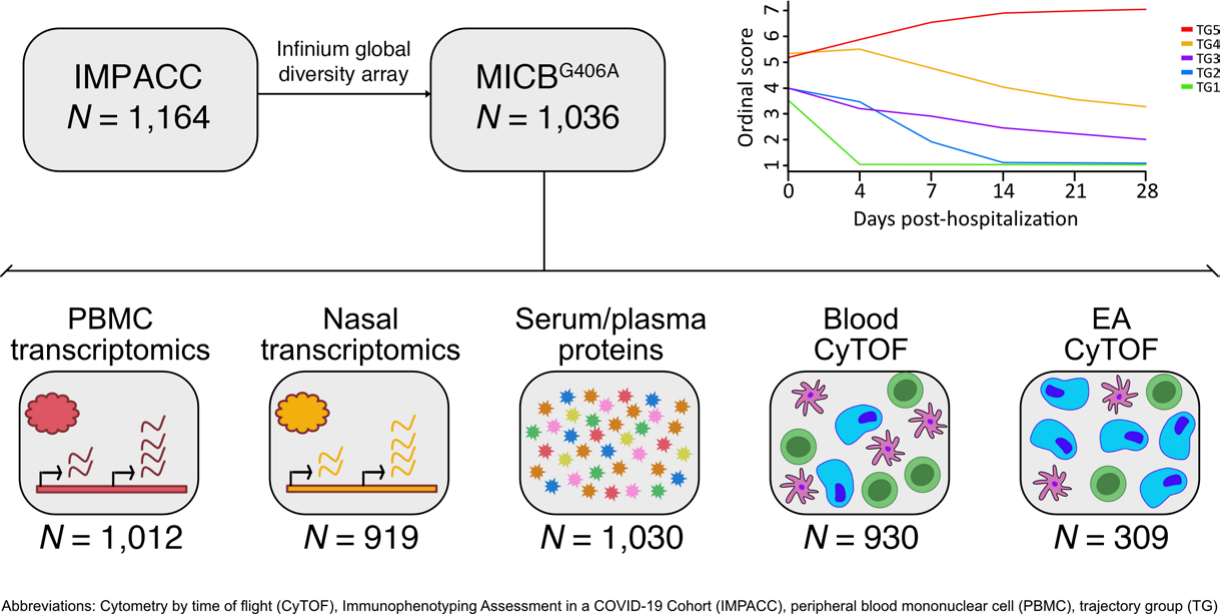
Schematic for the study and the nested subsets of assays available for immunophenotyping.

IMPACC (36) enrolled 1,164 unvaccinated patients hospitalized with SARS-CoV-2 infection across 20 US hospitals from May 2020 to March 2021, capturing patient demographics and COVID-19 outcome measures (37) and performing multi-omic phenotyping of patients (38). The *MICB^G406A^* genotype (rs1051788) was available for 1,036 patients from Infinium Global Diversity Array sequencing of DNA from peripheral blood. The majority of patients (54%) were homozygous for the major allele; 41% and 5% had 1 or 2 copies of the variant allele, respectively (Table 1). The frequency of homozygous MICB^G406A^ patients was lower than we have reported previously in the Validating Acute Lung Injury Markers for Diagnosis (VALID) cohorts of sepsis (*n* = 1,376, homozygous MICB^G406A^ = 12.0%) and ARDS (*n* = 733, homozygous MICB^G406A^ = 11.2%) (20) but similar to the Genome Aggregation Database (gnomAD, *n* = 151,970, homozygous MICB^G406A^ = 5.1%) reference dataset (39). As expected, the frequency of the *MICB^G406A^* variant allele varied across study sites and by self-reported ancestry. The variant allele frequency was higher in patients of non-Hispanic and Black/African American ancestry. Additional blood clinical measures were not associated with MICB^G406A^ polymorphism (Supplemental Data File 1; supplemental material available online with this article; https://doi.org/10.1172/jci.insight.191951DS1); however, we did find a significant association with asthma and nonasthma pulmonary disease. Figure 1 shows the design for the study and the nested subsets of assays available for immunophenotyping.

### MICB^G406A^ variant alleles are associated with reduced morbidity and mortality.

**Figure 2:**
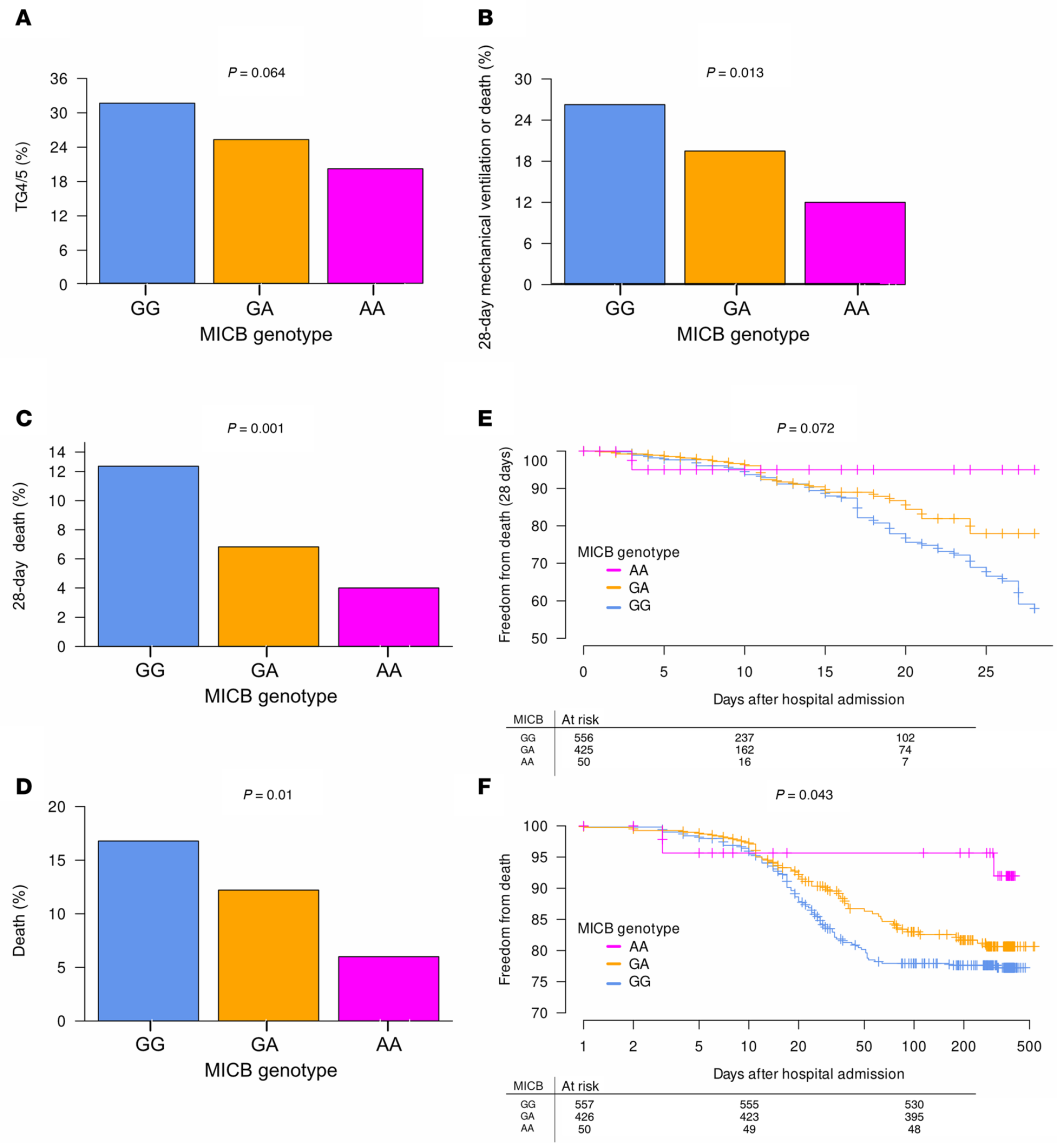
COVID-19 morbidity and mortality by MICB^G406A^ polymorphism. (**A**–**D**) Frequency of severe COVID-19 (trajectory group 4 or 5) (**A**), need for mechanical ventilation or death by 28-days post-admission (ordinal respiratory score ≥ 6) (**B**), death by 28-days post-admission (**C**), and death (**D**) at any point during the study by number of copies of the MICB^G406A^ variant allele were compared by binomial generalized logistic regression. (**E** and **F**) Survival analysis of death by 28 days after admission (**E**) or ever during the study (**F**) and number of copies of the MICB^G406A^ variant allele were compared by Cox proportional hazards models. Results for patients with no copies of the variant allele (GG) are shown in blue, 1 copy of the variant allele (GA) in orange, and 2 copies of the variant allele (AA) in magenta.

The primary COVID-19 metric used by this study is trajectory group (TG), defined by clustering of patients based on longitudinal dynamics of their ordinal respiratory score (37). TG1–TG3 patients had mild to moderate disease based on hospital stay and level of respiratory support, while severe disease was characterized by longer hospitalizations and prolonged respiratory support requirements (TG4) or by death within 28 days (TG5). We found no differences in baseline TG score across genotypes (adjusted *P* [*P*_adj_]= 0.919), but *MICB^G406A^* conferred reduced odds of severe COVID-19 prior to adjustment (Figure 2A; odds radio [OR] = 0.73, 95% CI = 0.58–0.93, *P* = 0.01, *P*_adj_ = 0.064), defined as patients in TG4 or TG5. Increasing copies of the MICB^G406A^ variant allele were associated with reduced cumulative odds for mechanical ventilation or death by day 28 (Figure 2B). Compared with MICB major allele, participants homozygous for MICB^G406A^ demonstrated 34% reduced cumulative odds for mechanical ventilation or death (OR = 0.66, 95% CI = 0.51–0.85, *P* = 0.002, *P*_adj_ = 0.013). Increasing copies of the *MICB^G406A^* variant allele were also associated with reduced odds of death, by day 28 (Figure 2C; OR = 0.53, 95% CI = 0.35–0.77, *P* = 0.002, *P*_adj_ = 0.001) or ever in the study (Figure 2D; OR = 0.65, 95% CI = 0.47–0.89, *P* = 0.008, *P*_adj_ = 0.010). Cox proportional hazards models similarly demonstrated reduced risk for death with increasing copies of the *MICB^G406A^* variant allele, either by day 28 prior to adjustment (Figure 2E; OR = 0.63, 95% CI = 0.43–0.92, *P* = 0.018, *P*_adj_ = 0.072) or ever in the study (Figure 2F; OR = 0.73, 95% CI = 0.54–0.98, *P* = 0.034, *P*_adj_ = 0.043). Consequently, the median survival for carriers of 2 copies of the *MICB^G406A^* variant allele was 313 days (interquartile range [IQR], 7–377 days) as compared with 41 days (IQR, 6–361 days) for single *MICB^G406A^* variant allele carriers or 61 days (IQR, 9–366 days) for patients homozygous for the major allele. However, these data are provisional, given incomplete long-term follow-up times. Notably, increasing copies of the *MICB^G406A^* variant allele were still associated with reduced odds of death when using days from self-reported symptom onset, rather than hospital admission. This finding was consistent for both death by day 28 after admission (Supplemental Figure 1A; OR = 0.48, 95% CI = 0.30–0.76, *P*_adj_ = 0.005) or ever in the study (Supplemental Figure 1B; OR = 0.60, 95% CI = 0.43–0.86, *P*_adj_ = 0.005).

### MICB^G406A^ variant alleles are not associated with SARS-CoV-2 antibodies or viral load.

**Figure 3:**
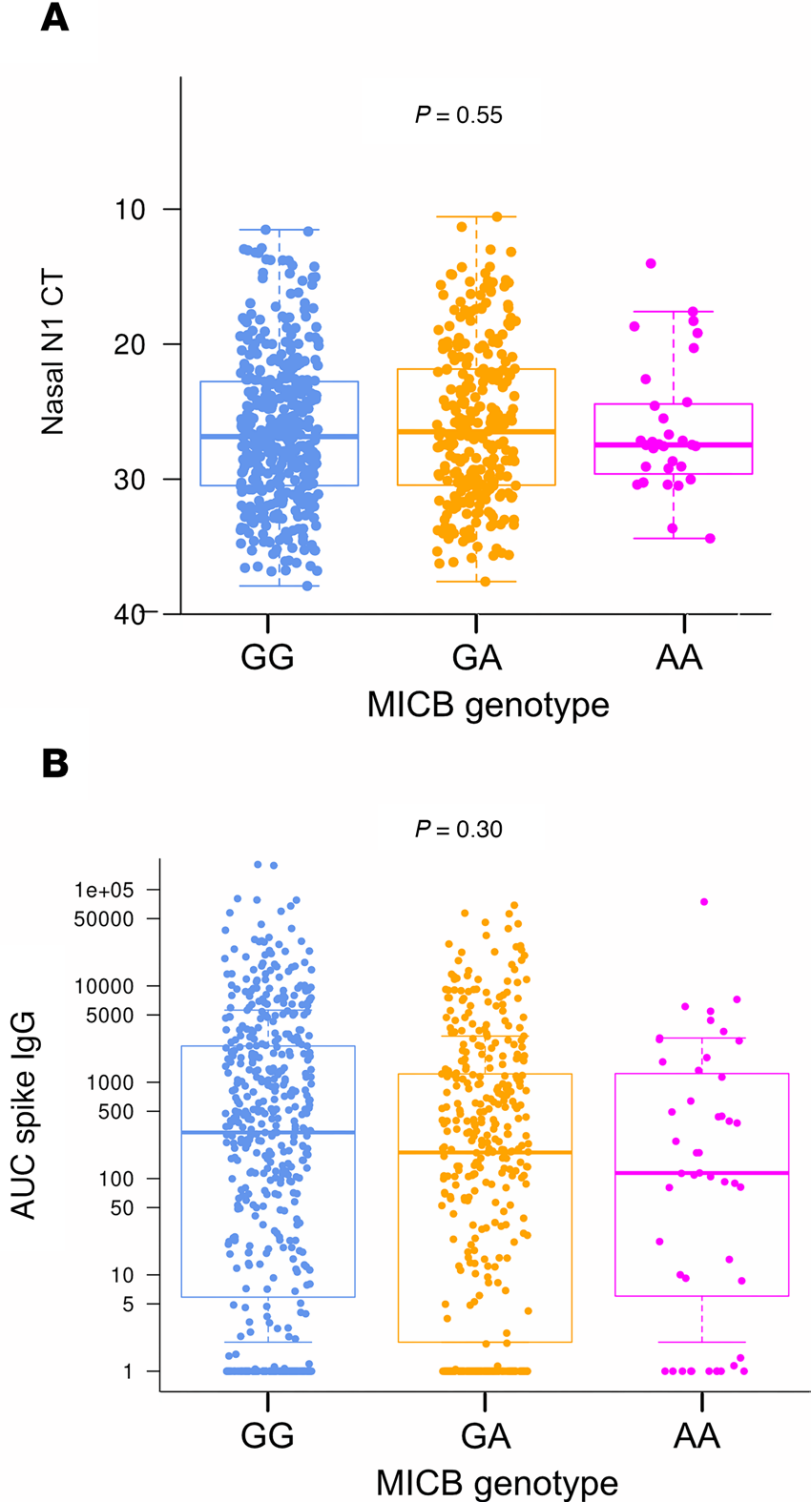
Nasal SARS-CoV-2 viral load and serum anti–Spike IgG by MICB^G406A^ polymorphism. (**A** and **B**) Visit 1 (**A**) nasal SARS-CoV-2 viral load (N1 CT value) (**A**) and serum anti–spike IgG levels (AUC) (**B**) by number of copies of the MICB^G406A^ variant allele were compared by linear regression. Results for patients with no copies of the variant allele (GG) are shown in blue, 1 copy of the variant allele (GA) in orange, and 2 copies of the variant allele (AA) in magenta.

Since increasing copies of the *MICB^G406A^* variant allele were protective against severe COVID-19, we next determined if this relationship was related to differences in either SARS-CoV-2 upper airway viral load or antibody titer. We found no association between *MICB^G406A^* variant alleles and SARS-CoV-2 nasal viral load determined by PCR at visit 1 (Figure 3A; *P* = 0.381, *P*_adj_ = 0.55) or longitudinally (Supplemental Figure 2A; *P* = 0.103, *P*_adj_ = 0.17). Serum anti–Spike IgG levels at visit 1 were lower in patients with increasing copies of the *MICB^G406A^* variant allele (Figure 3B; *P* = 0.023), but this did not reach our prespecified level of statistical significance (*P*_adj_ = 0.30). Similarly, we found no difference in longitudinal anti–Spike IgG levels (Supplemental Figure 2B; *P* = 0.758, *P*_adj_ = 0.76). Overall, these data show no association between MICB genotype and viral replication or between MICB genotype and humoral immune competence, suggesting an alternate mechanism of action.

### Relationship between MICB^G406A^ variant alleles and MICB expression varies by site.

**Figure 4:**
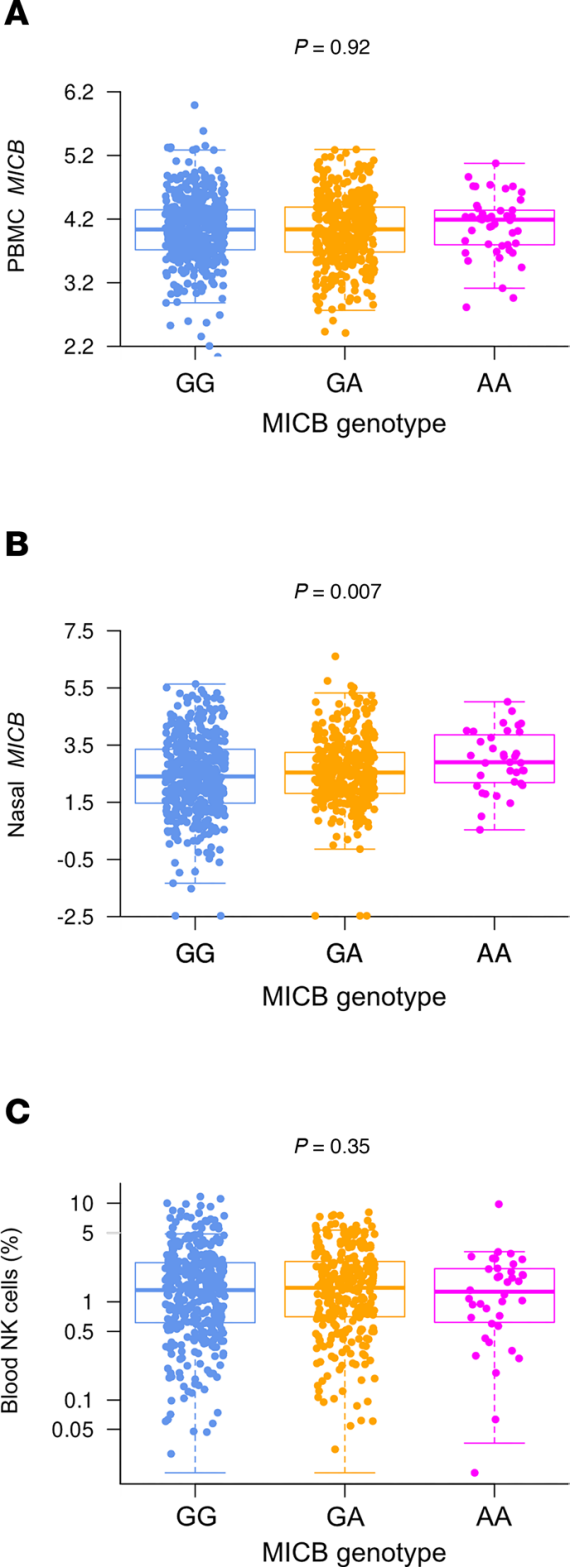
MICB gene expression in PBMC and the upper airway by MICB^G406A^ polymorphism. (**A** and **B**) Visit 1 MICB gene expression in PBMC (**A**) and nasal transcriptomics (**B**) by number of copies of the MICB^G406A^ variant allele was compared by linear regression. (**C**) Blood NK cell frequency at visit 1 by number of copies of the MICB^G406A^ variant allele were compared by linear regression. Results for patients with no copies of the variant allele (GG) are shown in blue, 1 copy of the variant allele (GA) in orange, and 2 copies of the variant allele (AA) in magenta.

We have previously reported that the *MICB^G406A^* variant allele in vitro is associated with reduced expression of MICB on primary human tracheal epithelial cells (20). Therefore, we compared *MICB* gene expression in this cohort by *MICB^G406A^* genotype. In PBMC transcriptomics, while *MICB* gene expression was higher in patients who died by day 28 (*P*_adj_ = 0.026) and trended similarly for TG45 patients (*P*_adj_ = 0.166), *MICB* expression did not differ with number of the *MICB^G406A^* variant allele (Figure 4A; *P* = 0.913, *P*_adj_ = 0.92). In nasal transcriptomics, *MICB* gene expression was higher in TG4–TG5 patients and those who died within day 28 (both *P*_adj_ = 0.006) and was also higher with increasing number of the *MICB^G406A^* variant alleles (Figure 4B; *P* = 0.007, *P*_adj_ = 0.007).

We have previously shown that the *MICB^G406A^* variant allele is associated with reduced frequency and maturity of NK cells in BAL fluid (20). Therefore, we compared the frequency and phenotype of NK cells by *MICB^G406A^* genotype in this cohort. In blood CyTOF data, there was no difference in frequency of NK cells at baseline (Figure 4C; *P* = 0.971, *P*_adj_ = 0.26) or longitudinally (Supplemental Figure 3; *P* = 0.51, *P*_adj_ = 0.35). This was also true for frequencies of immature (CD16^–^, *P* = 0.16 and *P* = 0.21) and mature (CD16^+^, *P* = 0.89 and *P* = 0.67) NK cells. In endotracheal aspirate CyTOF data, due to the low number of samples from patients with AA genotype, we compared NK cells between patients homozygous for the major allele and those with any variant alleles. As seen in blood, there was no difference in frequency of NK cells in samples collected closest to start of intubation (*P* = 0.2) or longitudinally (*P* = 0.26).

### Decreased soluble inflammatory markers associated with MICB^G406A^ variant alleles.

**Figure 5:**
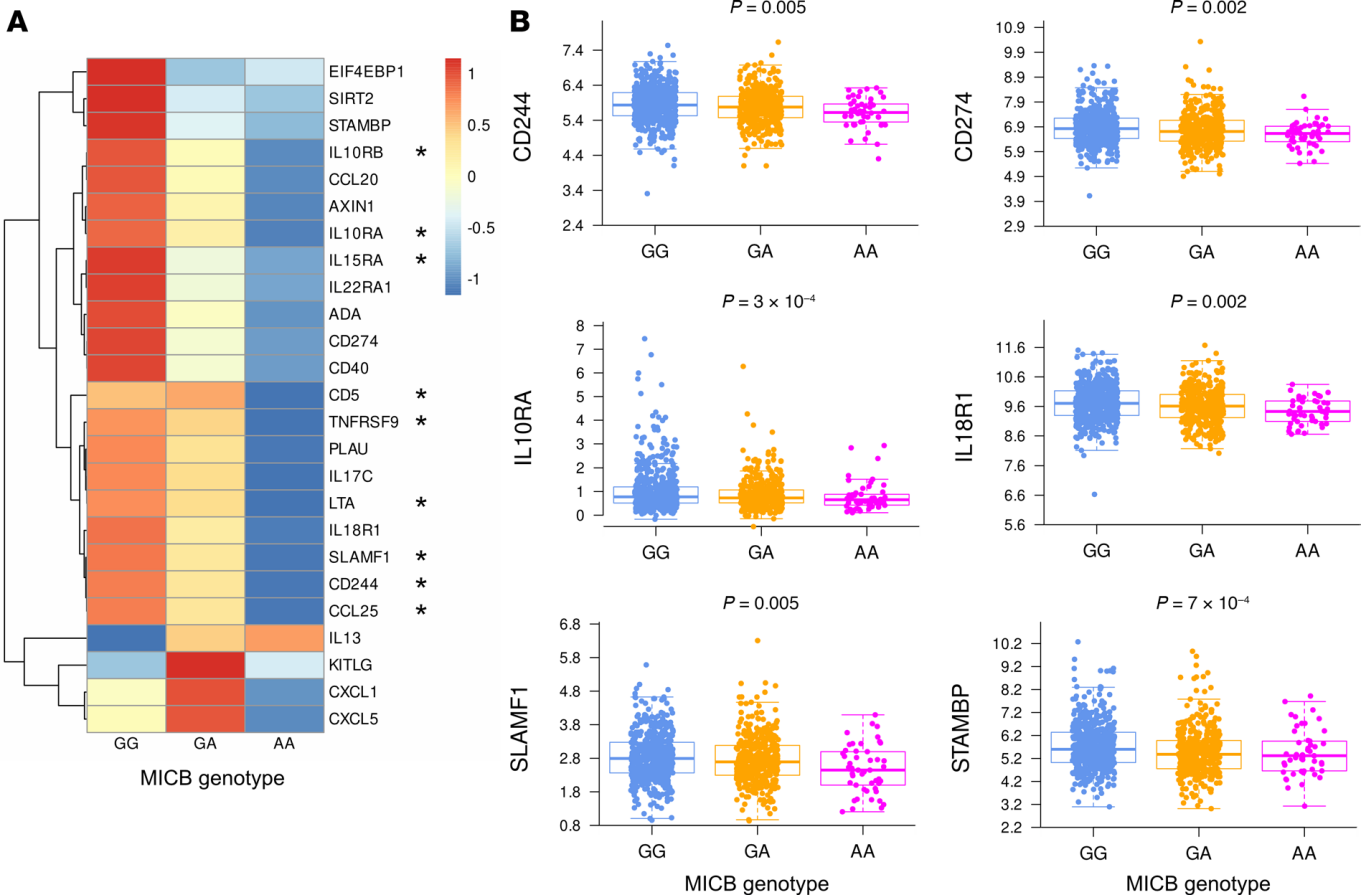
Serum proteins by MICB^G406A^polymorphism. Visit 1 serum protein levels by number of copies of the MICB^G406A^ variant allele were compared by linear regression. (**A** and **B**) Z-scaled serum levels by MICB^G406A^ genotype for all proteins with FDR ≤ 0.05 (**A**) and unscaled serum levels for key proteins (**B**) are shown. Analytes significantly associated with *MICB^G406A^* genotype after adjusting for baseline disease severity are indicated by asterisks in **A**. Results for patients with no copies of the variant allele (GG) are shown in blue, 1 copy of the variant allele (GA) in orange, and 2 copies of the variant allele (AA) in magenta.

COVID-19 is associated with high levels of multiple proinflammatory proteins in peripheral blood; we therefore compared serum protein levels by *MICB^G406A^* genotype in this cohort. In serum analytes at visit 1, quantified by Olink (*n* = 92), there was a stepwise decrease in 21 inflammatory mediators with increasing copies of the *MICB^G406A^* variant allele (Figure 5A and Supplemental Figure 4A; each analyte FDR-adjusted *P* < 0.05). Of these 21 proteins, 9 remained significantly associated with *MICB^G406A^* genotype after adjusting for baseline disease severity. These included markers of lymphocyte signaling, such as CD244, CD274, IL-17C, and receptors for IL-10, IL-15, and IL-18 (Figure 5B). Across all time points, only 2 analytes were associated with *MICB^G406A^* genotype (Supplemental Figure 4, B and C). CXCL8 (*P* = 0.004, *P*_adj_= 0.008) and TGF-α (*P* = 0.008, *P*_adj_ = 0.023) both decreased longitudinally in patients with AA genotype, while increasing marginally in patients with GG or GA genotypes.

### Reduced cellular activity and altered immune responses with MICB^G406A^ variant allele.

**Figure 6:**
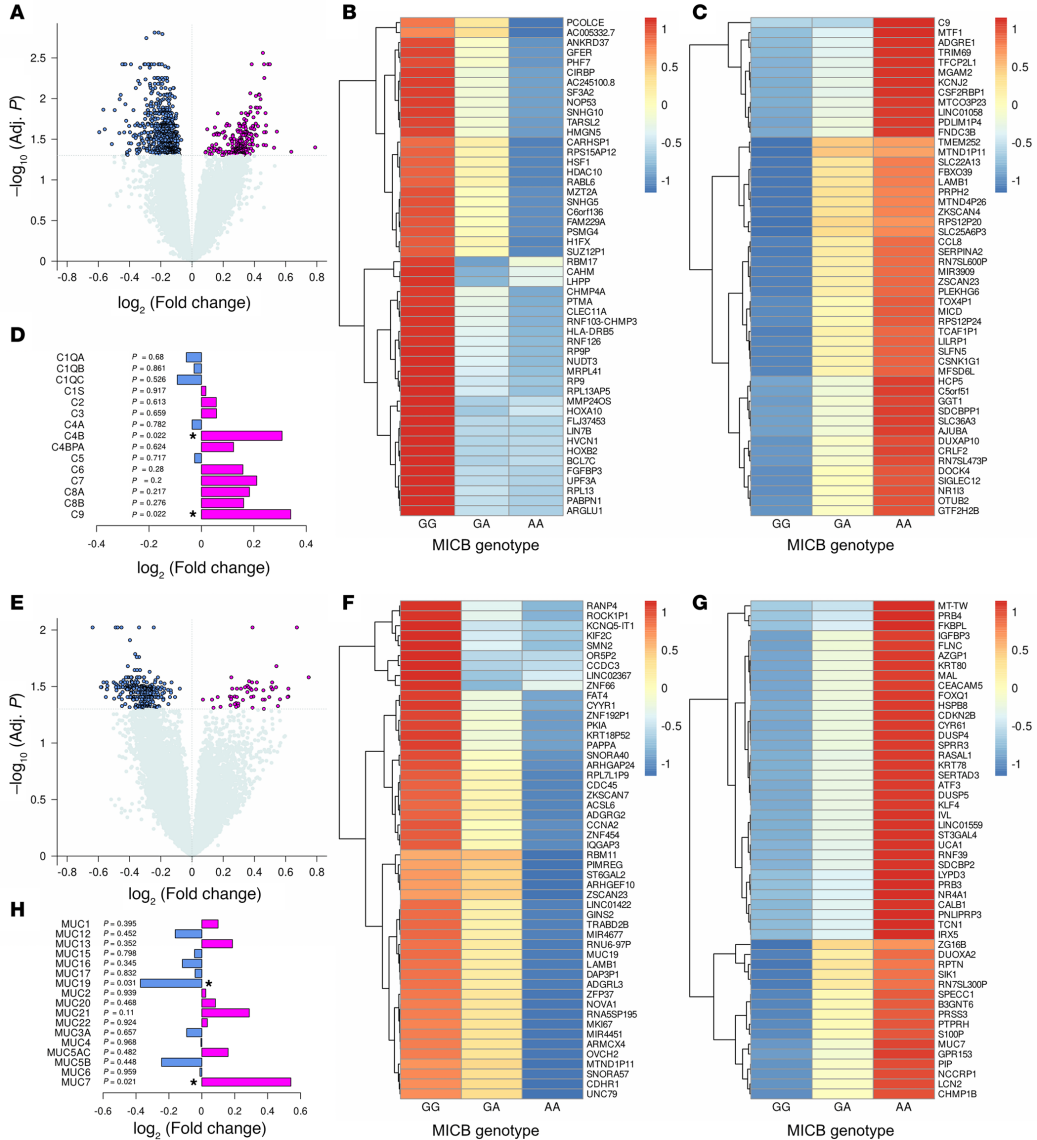
PBMC and upper airway gene expression by MICB^G406^ polymorphism. Visit 1 PBMC gene expression by number of copies of the MICB^G406A^ variant allele were compared by linear regression with the limma package. (**A**) Log-transformed fold-change with increasing copies of the variant allele against *P* values corrected for multiple comparisons is shown. (**B** and **C**) Z-scaled expression by MICB^G406A^ genotype for the top downregulated (**B**) and upregulated (**C**) genes is shown. (**D**) Log-transformed fold-change for all complement factors is shown with FDR-corrected *P* values. Visit 1 upper airway gene expression by number of copies of the MICB^G406A^ variant allele were compared by linear regression with the limma package. (**E**) Log-transformed fold-change with increasing copies of the variant allele against P value corrected for multiple comparisons is shown. (**F** and **G**) Z-scaled expression by MICB^G406A^ genotype for the top downregulated (**F**) and upregulated (**G**) genes is shown. (**H**) Log-transformed fold-change for all complement factors is shown with FDR-corrected P-values. Results for patients with no copies of the variant allele (GG) are shown in blue, 1 copy of the variant allele (GA) in orange, and 2 copies of the variant allele (AA) in magenta. In **A**, **D**, **E**, and **H**, blue and magenta indicate genes downregulated and upregulated with increasing copies of the MICB^G406A^ variant allele, respectively.

To further explore the mechanistic relationship between *MICB^G406A^* genotype and COVID-19, we performed differential gene expression analyses from PBMC and nasal transcriptomic data. In PBMC transcriptomics at visit 1 (Figure 6A), we identified 244 and 647 genes significantly upregulated and downregulated, respectively, with increasing copies of the *MICB^G406A^* variant allele, after adjustment for multiple testing. The downregulated genes (Figure 6B) were mostly related to cellular activity, including transcription, translation, and cell division, supported by GO term enrichment analysis (Supplemental Data File 1). These genes also highlighted autophagy and/or heat-shock responses (*ANKRD37*, *HSF1*) and type-1 IFN signaling (*RPL13*, *PTMA*). While the upregulated genes (Figure 6C) were more heterogeneous, some of the upregulated genes were involved in immune signaling, such as *CRLF2*, *SLFN5*, *C9*, *CCL8*, *OTUB2*, and *TRIM69*. Beyond *C9*, multiple complement factors were upregulated with increasing copies of the *MICB^G406A^* variant allele (Figure 6D), most notably for *C4B*. To confirm the upregulation of complement factor C9, we compared complement protein levels in plasma for these patients. C9 was nonsignificantly higher with increasing copies of the *MICB^G406A^* variant allele (*P* = 0.08). C1QA (*P* = 0.002) and C4B (*P* = 0.0002) were both significantly higher, while C5 (*P* = 0.007) and C6 (*P* = 0.007) were significantly lower.

In nasal transcriptomics at visit 1 (Figure 6E), we identified 54 and 328 genes significantly upregulated and downregulated, respectively, with increasing copies of the *MICB^G406A^* variant allele, after adjustment for multiple testing. The downregulated genes (Figure 6F) were again mostly related to the cell cycle and proliferation, supported by GO term enrichment (Supplemental Data File 2). The upregulated genes (Figure 6G) were related to antiinflammatory function and lymphocyte dysfunction, such as *ATF3*, *DUSP4* and *DUSP5*, *CEACAM5*, *NR4A1*, and *RASAL1*. Additionally, multiple proline-rich proteins were observed. While the mucin *MUC7* was upregulated, expression of other mucins by *MICB^G406A^* genotype was variable (Figure 6H), with *MUC19* significantly downregulated.

### Absence of a hyperinflammatory subtype in patients homozygous for the MICB^G406A^ variant allele.

**Figure 7:**
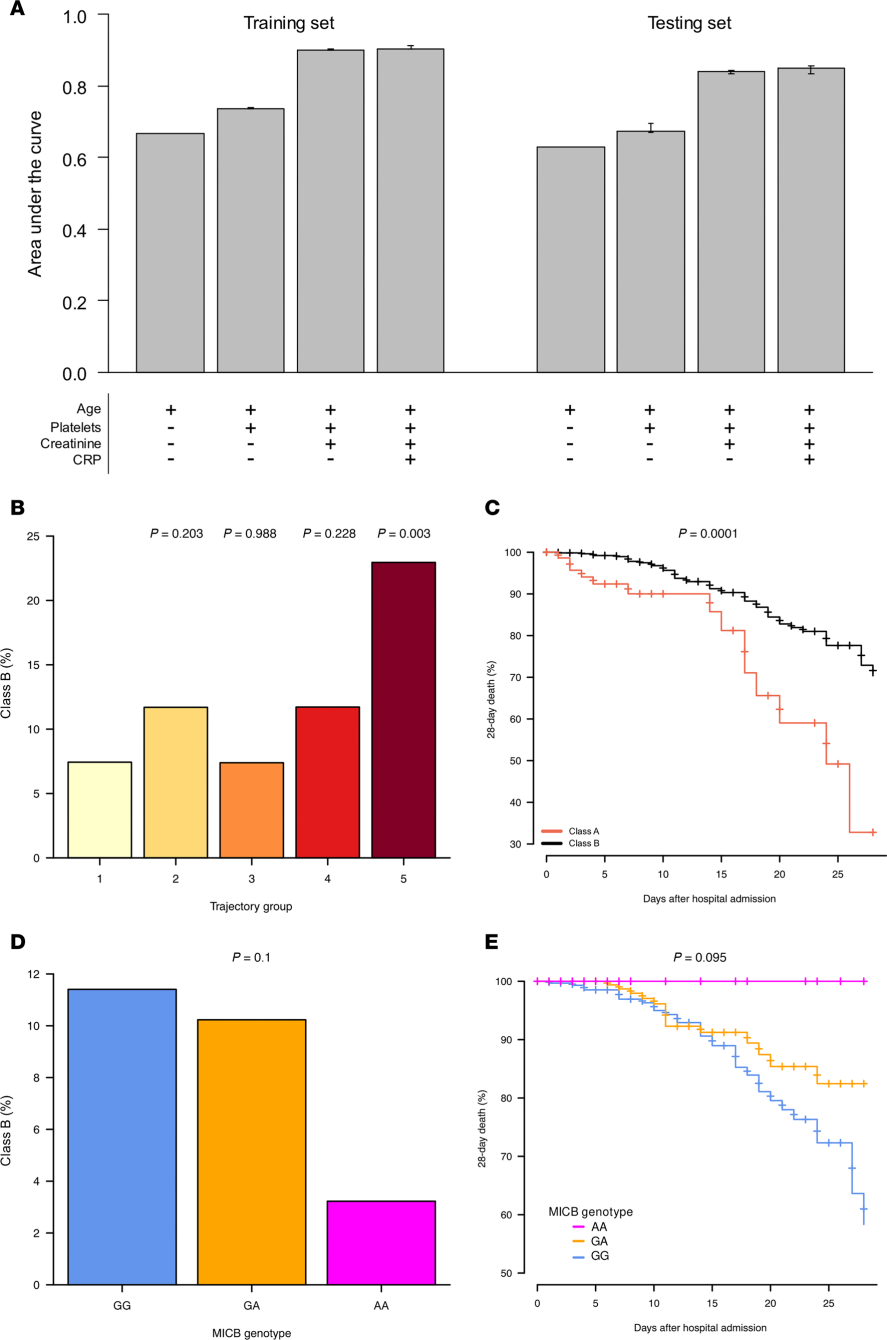
Hyperinflammatory subtype is associated with severe disease and decreasing copies of the MICB^G406A^ variant allele. (**A**) Odds of hyperinflamm atory subtype (Class B) by trajectory group was compared by binomial generalized logistic regression. (**B**) Survival analysis of death by 28 days after admission and inflammatory subtype was compared by Cox proportional hazards models. (**C**) Odds of Class B hyperinflammatory subtype by number of copies of the MICB^G406A^ variant allele was compared by binomial generalized logistic regression. (**D**) Survival analysis of death by 28 days after admission and number of copies of the MICB^G406A^ variant allele in patients not displaying the Class B hyperinflammatory subtype was compared by Cox proportional hazards models. (**E**) Results for patients with no copies of the variant allele (GG) are shown in blue, 1 copy of the variant allele (GA) in orange, and 2 copies of the variant allele (AA) in magenta.

A prior study of patients hospitalized with severe COVID-19, requiring at least 6 liters per minute (LPM) of supplemental oxygen up to invasive mechanical ventilation, used LCA of protein biomarkers and clinical variables to define 2 subtypes of severe COVID-19 (40). A hyperinflammatory subtype — characterized by persistent inflammation, evidence of endothelial and epithelial injury, and dysregulation of coagulation — was associated with twice the mortality rate of the less inflammatory subtype. Based on this LCA, we derived a clinical classifier model to identify patients with a similar hyperinflammatory subtype in our cohort, defined as Class B, and determine the association of this subtype with *MICB^G406A^* genotype. Figure 7A shows the cross-validated training and testing area under the curve (AUC) for each LCA with stepwise variable addition.

For TG1–TG4, between 7% and 12% of patients displayed the Class B subtype (Figure 7B). Odds of having the Class B profile were not significantly different for TG2–TG4 compared with TG1. Conversely, nearly 25% of patients in TG5 had the Class B profile, significantly increased compared with TG1 (*P*_adj_ = 0.003). Furthermore, the Class B subtype was associated with increased risk of death by day 28 (Figure 7C; HR = 3.09, 95% CI = 1.70–5.62, *P* = 0.0002, *P*_adj_ = 0.0001). This Class B profile was almost completely absent in patients with 2 copies of the *MICB^G406A^* variant allele, only 1 of 31 patients, and there was a nonsignificant trend toward reduced odds of having the Class B profile with increasing copies of the *MICB^G406A^* variant allele (Figure 7D; *P* = 0.248, *P*_adj_ = 0.1). Since there was only 1 patient with 2 copies of the *MICB^G406A^* variant allele and the Class B profile, we asked whether *MICB^G406A^* genotype was still associated with differences in outcome in patients lacking the Class B profile. Strikingly, even in the absence of the defined Class B profile, increasing copies of the *MICB^G406A^* variant allele were also associated with reduced risk of death by day 28 prior to adjustment (Figure 7E; HR = 0.49, 95% CI = 0.27–0.87, *P* = 0.015, *P*_adj_ = 0.095).

## Discussion

This study of patients hospitalized with SARS-CoV-2 infection found that increasing copies of the *MICB^G406A^* variant allele were associated with protection against severe disease, mechanical ventilation, and mortality, despite no detected differences in SARS-CoV-2 viral load or anti–Spike IgG titer. Patients with *MICB^G406A^* variant alleles had globally reduced inflammation, which may be driven by decreased activation of NKG2D-associated pathways, as well as specific immune dysregulation in both the periphery and upper airway, including altered profiles of peripheral complement and nasal mucins. Lastly, an adaptation of a previously published hyperinflammatory subtype of severe COVID-19, associated with increased mortality, was almost entirely absent in patients homozygous for the *MICB^G406A^* variant allele (41, 42).

Increasing copies of the *MICB^G406A^* variant allele were associated with reduced risk of both severe disease and mortality due to COVID-19 in this cohort, and the absence of a hyperinflammatory subtype that predicted increased risk of mortality. Strikingly, even in patients without this hyperinflammatory profile, increasing copies of the variant allele were still associated with prolonged survival. This latter finding suggests that NK cell activation via stress recognition may constitute a previously unrecognized, separate mechanism of ALI, not associated with accepted markers of severe disease such as age and baseline platelet count, creatinine, and C-reactive protein levels. Given that MICB is a stress-induced ligand of the NK activating receptor NKG2D, it is likely that this effect is driven by NK cells or T cells bearing NK cell receptors. However, the relationship of NK cells with SARS-CoV-2 infection and COVID-19 is complicated (43, 44). Early after infection, NK cells are reduced in the periphery, most clearly in severe disease, and higher frequency correlates with faster viral clearance. Conversely, NK cells are more frequently identified in the lungs of patients with severe disease and upregulate markers of activation and proliferation. It is likely, therefore, that NK cells are involved in restricting local spread of SARS-CoV-2 infection, while also exacerbating immunopathology through their hyperinflammatory activity. Notably, while NK cells can kill SARS-CoV-2–infected cells in vitro, the virus can evade this fate by downregulating ligands of NKG2D, including MICB, although this effect was not observed within the first 24 hours after infection (45). If this evasion tactic occurs in vivo, it suggests a model whereby patients with increasing copies of the *MICB^G406A^* variant allele, which leads to reduced expression of MICB on pulmonary airway cells, would have less NK cell–mediated killing of SARS-CoV-2–infected or transformed cells, potentially limiting the immunopathology of COVID-19 that drives lung injury and mortality. In support of this, despite the protective effect of *MICB^G406A^* for COVID-19, we found no significant difference in baseline or longitudinal SARS-CoV-2 nasal viral load.

While *MICB^G406A^* variant alleles were associated with reduced levels of multiple proinflammatory proteins in serum, we found that complement proteins in plasma were less consistent. C5 and C6 were both decreased, but C1QA and C4B were both increased. Much like NK cells, the complement system appears to play a multifaceted role in SARS-CoV-2 infection and COVID-19, likely dependent on differential dynamics over time after infection (46, 47). Early activation of complement likely supports clearance of the virus, while continued activation in severe disease may promote tissue injury. Notably, the alternative and lectin pathways of complement activation have been more frequently observed in severe COVID-19. It is possible, therefore, that the balance of C1QA over complement proteins involved in the formation of the membrane attack complex (MAC) in *MICB^G406A^* patients, indicates that activation of the classical pathway is less pathogenic in this context. In support of this, classical pathway component C1q, as well as the MAC inhibitor C4BP, have been shown in vitro to bind directly to SARS-CoV-2, limiting viral entry and induction of proinflammatory cytokines (48). Interestingly, an HLA-B*57:01–related haploblock found in HIV Elite Controllers, individuals positive for HIV who maintain low viral loads without treatment, was associated with upregulation of C4A but with downregulation of both MICB and C4B (49). Therefore, it is plausible that a potential connection between MICB and complement is due to genetic linkage, rather than a direct relationship.

This study also found differences in MICB and mucin expression in the upper airway by *MICB^G406A^* genotype. Notably, *MICB* transcripts were increased in homozygous *MICB^G406A^* participants, which is concordant with data available in the Gene Tissue Expression (GTEx) project dataset (50). This potentially reflects a negative feedback loop, as we previously observed reduced MICB protein on the surfaces of cells with the same genotypes (20). In the mucin domain, *MUC7* and *MUC19* were up- and down-regulated, respectively, with increasing copies of the protective variant allele. Mucins are a major component of saliva with distinct functions in immune defense against pathogens; however, they are also induced by and can contribute to proinflammatory responses (51). Therefore, it is not surprising that they have been implicated in both inhibiting SARS-CoV-2 infectivity (52, 53) and promoting COVID-19 immunopathology. MUC7 is induced by many cytokines (54), as well as damage-associated molecular patterns (DAMPs) and pathogen-associated molecular patterns (PAMPs), and can inhibit HIV infectivity (55, 56). Interestingly, genetic studies of MUC7 suggest it is a key target of salivary mucin evolutionary adaptations (57), likely due to pathogen exposure. Additionally, a study of asymptomatic and symptomatic SARS-CoV-2 cases found that salivary MUC7 was downregulated compared with uninfected controls (58). While MUC19 is not as well studied, it is also induced by inflammation and proinflammatory molecules (59), particularly TNF-α. MUC19 can promote bacterial clearance (60) but has been implicated in immunopathology of metapneumovirus infection (61). Taken together, these data suggest that a component of the protective effect of *MICB^G406A^* may be related to differential expression of functionally distinct mucins. In support of a potential link between mucins and MICB, a transmembrane component of MUC1 (MUC1-C) has previously been shown to repress in vitro expression of both MICA and MICB (62) through epigenetic modifications, limiting NK cell–mediated killing. Finally, alterations to MUC5 in the lung epithelium have been implicated in both fatal asthma (63) and idiopathic pulmonary fibrosis (64). These mucin dynamics were observed in the nasal epithelium, which may be discordant to the mucin expression within the lower respiratory tract.

This study has several notable strengths. This investigation was conducted with rigor across 20 hospitals. We were able to adjust for relevant confounders, thanks to a collection of detailed demographic and immunologic information on the cohort. However, this study also has some limitations. Many of the patients with respiratory failure within this study have ARDS; however, we were unable to clinically adjudicate ARDS based on limitations in the data collected at the time of the study. Thus, we employed an imprecise term, ALI, to describe the underling pathophysiologic process. We had a relatively low frequency of patients homozygous for the *MICB^G406A^* variant allele, ~5% of the cohort. While even 1 copy of the variant allele was clearly beneficial compared with no copies, there was a stepwise reduction in risk for severe COVID-19 with increasing copies of the variant allele. It is worth noting that this study occurred in hospitalized patients and that AA carriers may be underrepresented given reduced severity, biasing our study against observing significance. In support of this, a meta-analysis of COVID-19 GWAS (65) showed a trend toward reduced risk of both hospitalization and critical disease in patients with the variant allele. However, additional investigations applying mendelian randomization in a better powered cohort may more thoroughly elucidate this effect. Future studies should include greater representation of patients homozygous for the variant allele to explore the effect of 1 versus 2 copies. Additionally, despite the multi-omic immune profiling incorporated in our analysis, the lack of lung tissue samples and somewhat limited profiling of NK cells, both peripherally and in the tissue, reduced our ability to precisely define the cellular mechanism for the observed results. Furthermore, lower airway samples were only collected from intubated patients, and AA carriers were underrepresented in this group. In addition, this polymorphism may affect T cell and NK cell activation or function, which we did not measure. Lastly, this study focused on immune responses early after admission to the hospital, due to missingness in longitudinal sampling and bias toward increased sampling of surviving patients. In the future, demonstrating MICB-driven differences in abundance and functionality of NK cells in the lung longitudinally during COVID-19, ideally starting from the onset of symptoms, would help develop interventions that can exploit this pathway in preventing the most critical outcomes. Given the lack of association with SARS-CoV-2 infectious burden, it would also be of interest to study the effect of the polymorphism in other settings of infectious ALI to confirm the pathogen nonspecificity of its effect.

In summary, this study identified increasing copies of *MICB^G406A^* variant alleles as a potentially novel predictor of reduced risk for severe outcomes of COVID-19 in patients hospitalized with SARS-CoV-2 infection, independent of nasal viral control and peripheral antibody responses. Given the known effect of *MICB^G406A^* polymorphism on NK cell activity in the context of lung injury, targeting this pathway may be a practical intervention for limiting ALI in diverse contexts.

## Methods

### Sex as a biological variable.

Our study included both female and male participants. The cohort analyzed herein included 400 females (38.6%) and 636 males (61.4%). There was no significant association between sex and *MICB* polymorphism examined in this study (*P* = 0.237). Sex was included as a covariate in all analyses.

### IMPACC.

IMPACC, which consisted of participants from 20 hospitals linked to geographically diverse academic institutions across the United States, enrolled 1,164 unvaccinated patients hospitalized with symptoms or signs of COVID-19 between May 5, 2020, and March 19, 2021 (36, 37). To be included in the study, these patients needed to have their SARS-CoV-2 infection confirmed by RT-PCR. The comprehensive study design, the schedule for collecting clinical data and biological samples, and the participants’ demographic details have been previously outlined (36–38). In brief, detailed clinical evaluations and samples (nasal, blood, and endotracheal aspirates [EA] for intubated participants only) were gathered within 72 hours of hospitalization (visit 1), on days 4, 7, 14, 21, 28, 90, 180, and 360 following hospital admission (visits 2–6), and 3, 6, 9, and 12 months after discharge (38). Whole blood was collected, and peripheral blood mononuclear cells (PBMC) were isolated and cryopreserved as previously described (36).

### Host genotyping.

DNA was extracted, and samples were genotyped on the Illumina Global Diversity Array as previously described (38). For quality control of this data, we required that both samples and variants had genotyping rates > 95%, that variants had a minor allele frequency > 1%, and that variants adhered to Hardy-Weinberg equilibrium (HWE) (*P* < 1 × 10^–6^). Next, we removed outlier samples based on heterozygosity and missingness, excluded samples with clinical sex discordant with genetically inferred sex (assessed via X-chromosome homozygosity), filtered out samples exhibiting relatedness beyond a third-degree relationship (p-hat > 0.1875), and excluded samples with a unique admixture that lacked an appropriate case-control pairing. For this analysis, we extracted the MICB rs1051788 (NC_000006.11:g.31474000G>A) polymorphism, abbreviating minor allele A as *MICB^G406A^*. This SNP was available from the array without imputation. While this SNP was overall not under HWE, the deviation (D) was small (D = 0.016, 95% 95% CI = 0.005–0.027, *P* = 0.007) and was likely due to population structure. In support of this, when stratified by genetic ancestry, the SNP was under HWE for participants with American-European (*P* = 0.114) ancestry and European ancestry (*P* = 0.116) but not for participants with African ancestry (P =.027). Additionally, when stratified by disease severity, the SNP was under HWE for TG4–TG5 patients with severe disease (*P* = 0.231) but not TG1–TG3 patients with less severe disease (*P* = 0.012), suggesting that disease state also affected HWE in this cohort.

### Clinical outcome variables.

Longitudinal measures of an adaptation of the WHO 7-point severity ordinal scale (OS1, not hospitalized, no limitations; OS2, not hospitalized, activity limitations, or requires home O_2_; OS3, hospitalized, not requiring supplemental O_2_; OS4, hospitalized, requiring O_2_; OS5, hospitalized on non-invasive ventilation, or high-flow O_2_; OS6, hospitalized on invasive mechanical ventilation, and/or extracorporeal membrane oxygenation [ECMO]; OS7, death) over time were clustered into 5 treatment groups using group-based trajectory modeling, a likelihood-based approach commonly used to group time series of clinical data, as described previously (37). For the severity analysis, we defined participants with mild disease as those with TG1–TG3, and participants with severe disease as those with TG4–TG5, with TG5 representing all fatal cases within 28 days of admission. Mechanical ventilation was defined as a respiratory ordinal score of 6. Additionally, we compared patients with confirmed mortality at any time during the study or within 28 days of hospital admission against survivors. Cumulative odds of mechanical ventilation or death by day 28 was defined as either confirmed mortality within 28 days of hospital admission or mechanical ventilation any time within 28 days of hospital admission.

### Analysis of SARS-CoV-2 antibody titers.

Antibody levels against the recombinant SARS-CoV-2 Spike protein were measured in the blood using a research-grade ELISA, as described (37). Briefly, following heat inactivation at 56°C for 1 hour, serum samples were added to plates coated with RBD. Optical density (OD) was measured in a Synergy 4 (BioTek) plate reader at 490 nm. The AUC was calculated, considering 0.15 OD as the cutoff.

### Analysis of SARS-CoV-2 viral abundance.

SARS-CoV-2 viral abundance was calculated as log_10_ (rpM+1), where rpM is the reads per million of SARS-CoV-2 as measured by nasal metatranscriptomics (37). Briefly, alignments from nasal metatranscriptomics data were obtained from raw fastq files using the CZ-ID (Chan Zuckerberg ID [https://czid.org]) pipeline (66), which removes human sequences, followed by reference-based taxonomic alignment against the National Center for Biotechnology Information (NCBI) NT and nonredundant (NR) databases and assembly of reads matching each taxon.

### Analysis of serum inflammatory protein (Olink) data.

All samples were processed with the Olink multiplex assay inflammatory panels (Olink Proteomics), according to the manufacturer’s instructions and as previously described (38). This inflammatory panel included 92 proteins associated with human inflammatory conditions. Target protein quantification was performed by real-time microfluidic qPCR via the Normalized Protein Expression (NPX) manager software. Data were normalized using internal controls in every sample, interplate control and negative controls, and correction factor and expressed as log_2_ scale proportional to the protein concentration. For additional quality control, we set any NPX measurements below the assay’s limit of detection (LOD) to zero.

### Analysis of targeted plasma proteomics.

Plasma samples were processed and analyzed as previously described (38), using an LC system (Nexera Mikros, Shimadzu) equipped with a Capillary C18 column (0.2 × 100 mm, 2.7 μm particle diameter, Shimadzu) coupled online to an 8060 triple quadrupole mass spectrometer instrument (Shimadzu). Peptide intensities were calculated using Skyline software (v20.2.1.315) (67); means of the peptide intensities were used for protein abundances.

### Analysis of CyTOF data.

PBMCs and EAs were phenotyped on the Fluidigm Helios mass cytometer using distinct panels of surface and intracellular markers, and the cell types were annotated using an automated annotation pipeline as previously described (38). Prior to analysis, we removed cells identified as RBCs, multiplets, debris, and those that were not identifiable with high confidence. These counts were converted to proportions per sample, by dividing each cell type count by the total cell count.

### Analysis of PBMC and nasal transcriptomics.

RNA was extracted from PBMC, and inferior nasal turbinate swabs and gene expression levels were quantified by RNA-Seq as previously described (38). For all RNA-Seq analyses, we retained protein-coding genes that had a minimum of 10 counts in at least 20% of the samples. We calculated normalization factors to scale library sizes using the calcNormFactors function from the edgeR package v3.40.2, and we then normalized the gene counts using the voom function (normalize.method = “quantile”) from the limma package v3.46.0, fitted a linear model for the gene expression with lmFit function (default settings), calculated the empirical Bayes statistics with eBayes function (default settings), and calculated the *P* values for differential expression controlling for FDR.

### ARDS subtype analysis.

A prior study of patients hospitalized with severe COVID-19 (I-SPY COVID), requiring ≥ 6 LPM of supplemental oxygen, used LCA of protein biomarkers and clinical variables to define 2 potentially novel subtypes of severe COVID-19 (40). To similarly phenotype the patients in IMPACC, a parsimonious classifier model was trained in the I-SPY COVID cohort using phenotype-defining variables that were available in IMPACC, including age and baseline platelet count, creatinine, and C-reactive protein levels. The variables were sequentially entered into stepwise logistic regression models to predict probability of phenotype membership. Model performance was assessed by calculating the area under the receiver operating characteristic curves, the Akaike information criteria (AIC), and the Youden Index. The best-performing model was used to derive the classifier regression function and applied to patients in IMPACC. Patients with a classifier predicted probability of ≥ 0.5 were designated as Class B.

### Statistics.

For all analyses, unless otherwise stated, *MICB^G406A^* variant allele count was treated as the independent variable and assuming additive genetic effect. Visit 1 (baseline) analyses were modeled as *clinical outcome-Y* on *MICB^G406A^* variant allele count by binomial logistic regression. For longitudinal analyses, we used generalized linear mixed effect models through R package lme4 (68), including patient ID as a random effect. Models were considered significant for *P* < 0.05. Survival and 28-day survival were assessed by Cox proportional hazards models. Significance was determined by the likelihood ratio test, and models were considered significant for *P* < 0.05. All analyses were performed unadjusted (*P*) as a reference and adjusted for age, sex, ancestry as captured by the first 3 principal components, and study site (*P*_adj_). Statistical model covariates were selected based on hypotheses of the causal structure in terms of potential confounders versus colliders and were also weighed against the risk of model overfitting (69).

### Study approval.

NIAID staff conferred with the Department of Health and Human Services Office for Human Research Protections (OHRP) regarding potential applicability of the public health surveillance exception (45CFR46.102; refs 1, 2) to the IMPACC study protocol. OHRP concurred that the study satisfied criteria for the public health surveillance exception, and the IMPACC study team sent the study protocol and participant information sheet for review and assessment to IRBs at participating institutions. Twelve institutions elected to conduct the study as public health surveillance, while 3 sites with prior IRB-approved biobanking protocols elected to integrate and conduct IMPACC under their institutional protocols (University of Texas at Austin, IRB 2020-04-0117; UCSF, IRB 20-30497; Case Western Reserve University, IRB STUDY20200573) with informed consent requirements. Participants enrolled under the public health surveillance exclusion were provided information sheets describing the study, samples to be collected, and plans for data deidentification and use. Those that requested not to participate after reviewing the information sheet were not enrolled. Participants did not receive compensation for study participation while hospitalized and subsequently were offered compensation during outpatient follow-up.

### Data availability.

Data files are available at ImmPort under accession no. SDY1760 and dbGAP accession no. phs002686.v1.p1. Values for all data points in graphs are reported in the Supporting Data Values file.

## Author contributions

Conceptualization was carried out by HP, OAA, and DRC. The cohort design was designed and recruited by the IMPACC investigator group. Data collection was conducted by the IMPACC investigator group, HTM, SKS, RRM, CC, HS, FK, CRL, OL, LRB, EM, LIRE, GAMC, RPS, CBC, EKH, ACS, DAH, DBC, FK, MAA, SCB, NIAH, JPM, CLH, WBM, BP, KCN, MMD, AFS, VS, MK, CB, DJE, JS, AO, BP, SHK, ADA, JDA, PMB, NR, MCA, SB, WE, and JRG. Methodology was developed by HP and DRC. Investigation was performed by HP, EFR, and DRC. Data analysis was handled by HP, NAL, EC, DD, and DRC. Visualization was managed by HP and DRC Funding acquisition was secured by CSC, OAA, and DRC. The original draft of the manuscript was written by HP and DRC. All authors participated in the review and editing of the manuscript.

## Supplementary Material

Supplemental data

Supporting data values

## Figures and Tables

**Table 1:**
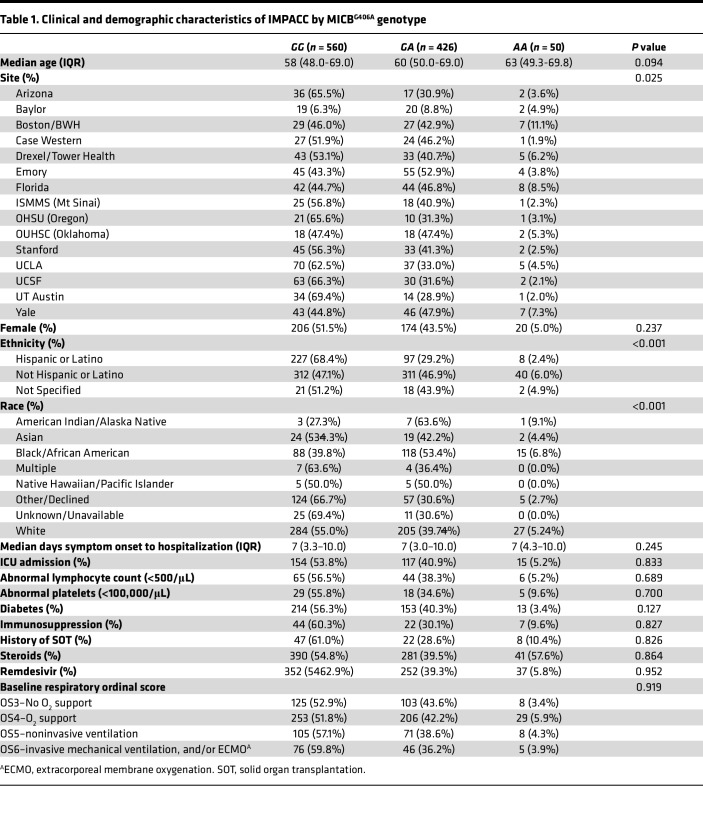
Clinical and demographic characteristics of IMPACC by MICB^G406A^ genotype

## Version 1 07/03/2025

In-Press Preview

## Version 2 08/08/2025

Electronic publication
