# Supplementary material for: MICBG406A polymorphism reduces risk of mechanical ventilation and death during viral acute lung injury
Source: JCI Insight. 2025 Jul 3;10(15):e191951. doi: 10.1172/jci.insight.191951 (PMC12333951; doi:10.1172/jci.insight.191951)
Supplement: Supplemental data [file jciinsight-10-191951-s215.pdf]

MICB^{G406A} **polymorphism reduces risk of mechanical ventilation and death during viral acute lung injury.**

Authors: Harry Pickering[1#], Narges Alipanah-Lechner[2], Ernie Chen[3], Dylan Duchen[3], Holden T. Maecker[4], Seunghee Kim-Sschulze[5], Ruth R. Montgomery[3], Chris Cotsapas[3], Hanno Steen[6], Florian Krammer[5], Charles R. Langelier[2], Ofer Levy[6], Lindsey R. Baden[7], Esther Melamed[8], Lauren I. R. Ehrlich[8], Grace A. McComsey[9], Rafick P. Sekaly[9], Charles B. Cairns[10], Elias K. Haddad[10], Albert C. Shaw[3], David A. Hafler[3], David B. Corry[11], Farrah Kheradmand[11], Mark A. Atkinson[12], Scott C. Brakenridge[12], Nelson I Agudelo Higuita[13], Jordan P. Metcalf[13], Catherine L. Hough[14], William B. Messer[14], Bali Pulendran[4], Kari C. Nadeau[4], Mark M. Davis[4], Ana Fernandez Sesma[5], Viviana Simon[5], Monica Kraft[15], Chris Bime[15], David J. Erle[2], Joanna Schaenmann[1], Al Ozonoff[6], Bjoern Peters[16], Steven H. Kleinstein[3], Alison D. Augustine[17], Joann Diray-Arce[3], Patrice M. Becker[17], Nadine Rouphael[18], Matthew C. Altman[19], Steve Bosinger[20], Walter Eckalbar[2], IMPACC Network[21], Carolyn S. Calfee[2], Oscar A. Aguilar[22,23], Elaine F. Reed[1], John R. Greenland[2,24], Daniel R. Calabrese[2,24]

Affiliations:

[1]Pathology and Laboratory Medicine, University of California Los Angeles, Los Angeles, CA

[2]Department of Medicine, University of California San Francisco, San Francisco, CA

[3]Yale School of Medicine, New Haven, CT

[4]Stanford University School of Medicine, Palo Alto, CA, USA

[5]Icahn School of Medicine at Mount Sinai, New York, NY, USA

[6]Precision Vaccines Program, Boston Children's Hospital, Harvard Medical School, Boston, MA, USA

[7]Brigham and Women's Hospital, Harvard Medical School, Boston, MA, USA

[8]The University of Texas at Austin, Austin, TX, USA

[9]Case Western Reserve University and University Hospitals of Cleveland, Cleveland, OH, USA

[10]Drexel University, Tower Health Hospital, Philadelphia, PA, USA
[11]Baylor College of Medicine and the Center for Translational Research on Inflammatory Diseases, Houston, TX, USA
[12]University of Florida, Gainesville, FL, USA
[13]Oklahoma University Health Sciences Center, Oklahoma City, OK, USA
[14]Oregon Health & Science University, Portland, OR, USA
[15]University of Arizona, Tucson AZ, USA
[16]La Jolla Institute for Immunology, La Jolla, CA, USA
[17]National Institute of Allergy and Infectious Diseases, National Institute of Health, Bethesda, MD, USA
[18]Emory School of Medicine, Atlanta, GA, USA
[19]Benaroya Research Institute, University of Washington, Seattle, WA
[20]Emory School of Medicine, Atlanta, GA
[21]The Immunophenotyping Assessment in a COVID-19 Cohort (IMPACC) Network is detailed in Supplementary Materials
[22]Department of Microbiology & Immunology, University of California San Francisco, San Francisco, CA
[23]Parker Institute for Cancer Immunotherapy, University of California San Francisco, San Francisco, CA
[24]Medical Service, San Francisco VA Health Care System, San Francisco, CA
[#]Corresponding author

Supplementary Materials

IMPACC Network

National Institute of Allergy and Infectious Diseases, National Institute of Health, Bethesda, MD 20814, USA: Patrice M. Becker, Alison D. Augustine, Steven M. Holland, Lindsey B. Rosen, Serena Lee, Tatyana Vaysman

Clinical and Data Coordinating Center (CDCC), Precision Vaccines Program, Boston Children's Hospital, Harvard Medical School, Boston, MA 02115, USA: Al Ozonoff, Joann Diray-Arce, Jing Chen, Alvin T. Kho, Carly E. Milliren, Annmarie Hoch, Ana C. Chang, Kerry McEnaney, Caitlin Syphurs, Brenda Barton, Claudia Lentucci, Maimouna D. Murphy, Mehmet Saluvan, Tanzia Shaheen, Shanshan Liu, Marisa Albert, Arash Nemati Hayati, Robert Bryant, James Abraham, Mitchell Cooney, Meagan Karoly

Benaroya Research Institute, University of Washington, Seattle, WA 98101, USA: Matthew C. Altman, Naresh Doni Jayavelu, Scott Presnell, Bernard Kohr, Tomasz Jancsyk, Azlann Arnett

La Jolla Institute for Immunology, La Jolla, CA 92037, USA: Bjoern Peters, James A. Overton, Randi Vita, Kerstin Westendorf

Knocean Inc. Toronto, ON M6P 2T3, Canada: James A. Overton

Precision Vaccines Program, Boston Children's Hospital, Harvard Medical School, Boston, MA 02115, USA: Ofer Levy, Hanno Steen, Patrick van Zalm, Benoit Fatou, Kinga K. Smolen, Arthur Viode, Simon van Haren, Meenakshi Jha, David Stevenson, Sanya Thomas, Boryana Petrova, Naama Kanarek

Brigham and Women's Hospital, Harvard Medical School, Boston, MA 02115, USA: Lindsey R. Baden, Kevin Mendez, Jessica Lasky-Su, Alexandra Tong, Rebecca Rooks, Michael Desjardins,

Amy C. Sherman, Stephen R. Walsh, Xhoi Mitre, Jessica Cauley, Xiaofang Li, Bethany Evans, Christina Montesano, Jose Humberto Licona, Jonathan Krauss, Nicholas C. Issa, Jun Bai Park Chang, Natalie Izaguirre

Metabolon Inc, Morrisville, NC 27560, USA: Scott R. Hutton, Greg Michelotti, Kari Wong

Prevention of Organ Failure (PROOF) Centre of Excellence, University of British Columbia, Vancouver, BC V6T 1Z3, Canada: Scott J. Tebbutt, Casey P. Shannon

Case Western Reserve University and University Hospitals of Cleveland, Cleveland, OH 44106, USA: Rafick-Pierre Sekaly, Slim Fourati, Grace A. McComsey, Paul Harris, Scott Sieg, George Yendewa, Mary Consolo, Heather Tribout, Susan Pereira Ribeiro

Drexel University, Tower Health Hospital, Philadelphia, PA 19104, USA: Charles B. Cairns, Elias K. Haddad, Michele A. Kutzler, Mariana Bernui, Gina Cusimano, Jennifer Connors, Kyra Woloszczuk, David Joyner, Carolyn Edwards, Edward Lee, Edward Lin, Nataliya Melnyk, Debra L. Powell, James N. Kim, I. Michael Goonewardene, Brent Simmons, Cecilia M. Smith, Mark Martens, Brett Croen, Nicholas C. Semenza, Mathew R. Bell, Sara Furukawa, Renee McLin, George P. Tegos, Brandon Rogowski, Nathan Mege, Kristen Ulring, Pam Schearer, Judie Sheidy, Crystal Nagle

MyOwnMed Inc., Bethesda, MD 20817, USA: Vicki Seyfert-Margolis

Emory School of Medicine, Atlanta, GA 30322, USA: Nadine Rouphael, Steven E. Bosinger, Arun K. Boddapati, Greg K. Tharp, Kathryn L. Pellegrini, Brandi Johnson, Bernadine Panganiban, Christopher Huerta, Evan J. Anderson, Hady Samaha, Jonathan E. Sevransky, Laurel Bristow, Elizabeth Beagle, David Cowan, Sydney Hamilton, Thomas Hodder, Amer Bechnak, Andrew Cheng, Aneesh Mehta, Caroline R. Ciric, Christine Spainhour, Erin Carter, Erin M. Scherer, Jacob

Usher, Kieffer Hellmeister, Laila Hussaini, Lauren Hewitt, Nina Mcnair, Susan Pereira Ribeiro, Sonia Wimalasena

Icahn School of Medicine at Mount Sinai, New York, NY 10029, USA: Ana Fernandez-Sesma, Viviana Simon, Florian Krammer, Harm Van Bakel, Seunghee Kim-Schulze, Ana Silvia Gonzalez-Reiche, Jingjing Qi, Brian Lee, Juan Manuel Carreño, Gagandeep Singh, Ariel Raskin, Johnstone Tcheou, Zain Khalil, Adriana van de Guchte, Keith Farrugia, Zenab Khan, Geoffrey Kelly, Komal Srivastava, Lily Q. Eaker, Maria C. Bermúdez-González, Lubbertus C.F. Mulder, Katherine F. Beach, Miti Saksena, Deena Altman, Erna Kojic, Levy A. Sominsky, Arman Azad, Dominika Bielak, Hisaaki Kawabata, Temima Yellin, Miriam Fried, Leeba Sullivan, Sara Morris, Giulio Kleiner, Daniel Stadlbauer, Jayeeta Dutta, Hui Xie, Manishkumar Patel, Kai Nie, Brian Monahan

Immunai Inc., New York, NY 10016, USA: Adeeb Rahman

Oregon Health & Science University, Portland, OR 97239, USA: William B. Messer, Catherine L. Hough, Sarah A.R. Siegel, Peter E. Sullivan, Zhengchun Lu, Amanda E. Brunton, Matthew Strand, Zoe L. Lyski, Felicity J. Coulter, Courtney Micheletti

Stanford University School of Medicine, Palo Alto, CA 94305, USA: Holden Maecker, Bali Pulendran, Kari C. Nadeau, Yael Rosenberg-Hasson, Michael Leipold, Natalia Sigal, Angela Rogers, Andrea Fernandes, Monali Manohar, Evan Do, Iris Chang, Alexandra S. Lee, Catherine Blish, Henna Naz Din, Jonasel Roque, Linda N. Geng, Maja Artandi, Mark M. Davis, Neera Ahuja, Samuel S. Yang, Sharon Chinthrajah, Thomas Hagan, Tyson H. Holmes, Koji Abe

David Geffen School of Medicine at the University of California Los Angeles, Los Angeles CA 90095, USA: Elaine F. Reed, Joanna Schaenman, Ramin Salehi-Rad, Adreanne M. Rivera, Harry Pickering, Subha Sen, David Elashoff, Dawn C. Ward, Jenny Brook, Estefania Ramires-Sanchez, Megan Llamas, Claudia Perdomo, Clara E. Magyar, Jennifer Fulcher

University of California San Francisco, San Francisco, CA 94115, USA: David J. Erle, Carolyn S. Calfee, Carolyn M. Hendrickson, Kirsten N. Kangelaris, Viet Nguyen, Deanna Lee, Suzanna Chak, Rajani Ghale, Ana Gonzalez, Alejandra Jauregui, Carolyn Leroux, Luz Torres Altamirano, Ahmad Sadeed Rashid, Andrew Willmore, Prescott G. Woodruff, Matthew F. Krummel, Sidney Carrillo, Alyssa Ward, Charles R. Langelier, Ravi Patel, Michael Wilson, Ravi Dandekar, Bonny Alvarenga, Jayant Rajan, Walter Eckalbar, Andrew W. Schroeder, Gabriela K. Fragiadakis, Alexandra Tsitsiklis, Eran Mick, Yanedth Sanchez Guerrero, Christina Love, Lenka Maliskova, Michael Adkisson, Aleksandra Leligdowicz, Alexander Beagle, Arjun Rao, Austin Sigman, Bushra Samad, Cindy Curiel, Cole Shaw, Gayelan Tietje-Ulrich, Jeff Milush, Jonathan Singer, Joshua J. Vasquez, Kevin Tang, Legna Betancourt, Lekshmi Santhosh, Logan Pierce, Maria Tecero Paz, Michael Matthay, Neeta Thakur, Nicklaus Rodriguez, Nicole Sutter, Norman Jones, Pratik Sinha, Priya Prasad, Raphael Lota, Sadeed Rashid, Saurabh Asthana, Sharvari Bhide, Tasha Lea, Yumiko Abe-Jones

Yale School of Medicine, New Haven, CT 06510, USA: David A. Hafler, Ruth R. Montgomery, Albert C. Shaw, Steven H. Kleinstein, Jeremy P. Gygi, Dylan Duchen, Shrikant Pawar, Anna Konstorum, Ernie Chen, Chris Cotsapas, Xiaomei Wang, Charles Dela Cruz, Akiko Iwasaki, Subhasis Mohanty, Allison Nelson, Yujiao Zhao, Shelli Farhadian, Hiromitsu Asashima, Omkar Chaudhary, Andreas Coppi, John Fournier, M. Catherine Muenker, Khadir Raddassi, Michael Rainone, William Ruff, Syim Salahuddin, Wade L. Shulz, Pavithra Vijayakumar, Haowei Wang, Esio Wunder Jr., H. Patrick Young, Albert I. Ko, Gisela Gabernet

Yale School of Public Health, New Haven, CT 06510, USA: Denise Esserman, Leying Guan, Anderson Brito, Jessica Rothman, Nathan D. Grubaugh, Kexin Wang, Leqi Xu

Baylor College of Medicine and the Center for Translational Research on Inflammatory Diseases, Houston, TX 77030, USA: David B. Corry, Farrah Kheradmand, Li-Zhen Song, Ebony Nelson

Oklahoma University Health Sciences Center, Oklahoma City, OK 73104, USA: Jordan P. Metcalf, Nelson I. Agudelo Higuita, Lauren A. Sinko, J. Leland Booth, Douglas A. Drevets, Brent R. Brown

University of Arizona, Tucson AZ 85721, USA: Monica Kraft, Chris Bime, Jarrod Mosier, Heidi Erickson, Ron Schunk, Hiroki Kimura, Michelle Conway, Dave Francisco, Allyson Molzahn, Connie Cathleen Wilson, Ron Schunk, Trina Hughes, Bianca Sierra

University of Florida, Gainesville, FL 32611, USA: Mark A. Atkinson, Scott C. Brakenridge, Ricardo F. Ungaro, Brittany Roth Manning, Lyle Moldawer

University of Florida, Jacksonville, FL 32218, USA: Jordan Oberhaus, Faheem W. Guirgis

University of South Florida, Tampa FL 33620, USA: Brittney Borresen, Matthew L. Anderson

The University of Texas at Austin, Austin, TX 78712, USA: Lauren I. R. Ehrlich, Esther Melamed, Cole Maguire, Dennis Wylie, Justin F. Rousseau, Kerin C. Hurley, Janelle N. Geltman, Nadia Siles, Jacob E. Rogers, Pablo Guaman Tipan

We thank the participants of the study for their voluntary enrollment and contribution of samples for this work. See the supplement for details on the IMPACC Network. We acknowledge the assistance of the following individuals: Sanya Thomas, Mitchell Cooney, Shun Rao, Sofia Vignolo, and Elena Morrocchi (all from the CDCC); Arash Naeim, Marianne Bernardo, Sarahmay Sanchez, Shannon Intluxay, Clara Magyar, Jenny Brook, Estefania Ramires-Sanchez, Megan Llamas, Claudia Perdomo, Clara E. Magyar, and Jennifer A. Fulcher (all from the David Geffen School of Medicine at UCLA); members of the UCLA Center for Pathology Research Services and the Pathology Research Portal; M. Catherine Muenker, Dimitri Duvilaire, Maxine Kuang, William Ruff, Khadir Raddassi, Denise Shepherd, Haowei Wang, Omkar Chaudhary, Syim Salahuddin, John Fournier, Michael Rainone, and Maxine Kuang (all from the Yale School of Medicine). We thank the leadership of Boston Children's Hospital including Drs. Wendy Chung, Gary Fleisher and

Kevin Churchwell for their support for the Precision Vaccines Program. Dr. Augustine's and Becker's co-authorship of this report does not necessarily represent the official views of the National Institute of Allergy and Infectious Diseases, the National Institutes of Health or any other agency of the United States Government.

Supplementary Data File 1. Additional patient clinical characteristics and comorbidities

	**GG (N=560)**	**GA (N=426)**	**AA (N=50)**	**P-value**
Abnormal CRP (>=10 mg/L)	234 (41.8%)	184 (43.2%)	20 (40.0%)	0.863
Abnormal D-dimer (>0.5 mg/L)	281 (50.2%)	213 (50.0%)	27 (54.0%)	0.794
Abnormal Troponin (>0.4 ng/mL)	43 (7.7%)	36 (8.5%)	5 (10.0%)	0.517
Abnormal Creatinine (>=1.5 mg/dL)	96 (17.1%)	64 (15.0%)	3 (6.0%)	0.065
Infiltrate on chest X-Ray	389 (74.5%)	287 (70.2%)	37 (77.1%)	0.408
Asthma	65 (11.7%)	75 (17.6%)	12 (24.0%)	0.001
Pulmonary disease (excludes asthma)	103 (18.4%)	94 (22.1%)	15 (30.0%)	0.033
Chronic kidney disease	81 (14.5%)	62 (14.6%)	12 (24.0%)	0.280

Chronic cardiac disease	135 (24.1%)	130 (30.5%)	14 (28.0%)	0.052

Supplementary Data File 2. PBMC and upper airway GO term enrichment analysis.

GO term enrichment analysis was performed on genes significantly upregulated (Cluster = “PBMC-Up”) or downregulated (Cluster = “PBMC-Down”) in PBMC of $MICB^{G406A}$ participants and genes significantly upregulated (Cluster = “Nasal-Up”) or downregulated (Cluster = “Nasal-Down”) in the upper airway. of $MICB^{G406A}$ participants.

Cluster	ONTOLOGY	ID	Description	GeneRatio	BgRatio	pvalue	p.adjust	qvalue	Count
PBMC-Up	MF	GO:0046943	carboxylic acid transmembrane transporter activity	4/46	164/18496	0.000728	0.059912	0.050922	4
PBMC-Up	MF	GO:0005342	organic acid transmembrane transporter activity	4/46	165/18496	0.000744	0.059912	0.050922	4
PBMC-Up	MF	GO:0008514	organic anion transmembrane transporter activity	4/46	242/18496	0.003032	0.16272	0.138304	4
PBMC-Up	MF	GO:0140375	immune receptor activity	3/46	147/18496	0.005816	0.230192	0.195652	3
PBMC-Up	MF	GO:0042379	chemokine receptor binding	2/46	74/18496	0.014588	0.230192	0.195652	2
PBMC-Up	MF	GO:0015171	amino acid transmembrane transporter activity	2/46	85/18496	0.018952	0.230192	0.195652	2
PBMC-Up	MF	GO:0004896	cytokine receptor activity	2/46	96/18496	0.023795	0.230192	0.195652	2

PBMC-Up	MF	GO:0015187	glycine transmembrane transporter activity	1/46	10/18496	0.0246	0.230192	0.195652	1
PBMC-Up	MF	GO:0015658	branched-chain amino acid transmembrane transporter activity	1/46	10/18496	0.0246	0.230192	0.195652	1
PBMC-Up	MF	GO:0019531	oxalate transmembrane transporter activity	1/46	10/18496	0.0246	0.230192	0.195652	1
PBMC-Up	MF	GO:0045294	alpha-catenin binding	1/46	10/18496	0.0246	0.230192	0.195652	1
PBMC-Up	MF	GO:0051864	histone H3K36 demethylase activity	1/46	10/18496	0.0246	0.230192	0.195652	1
PBMC-Up	MF	GO:0008271	secondary active sulfate transmembrane transporter activity	1/46	11/18496	0.027027	0.230192	0.195652	1
PBMC-Up	MF	GO:1990459	transferrin receptor binding	1/46	11/18496	0.027027	0.230192	0.195652	1
PBMC-Up	MF	GO:0004875	complement receptor activity	1/46	12/18496	0.029448	0.230192	0.195652	1

PBMC-Up	MF	GO:0009982	pseudouridine synthase activity	1/46	13/18496	0.031863	0.230192	0.195652	1
PBMC-Up	MF	GO:0016755	aminoacyltransferase activity	4/46	494/18496	0.033942	0.230192	0.195652	4
PBMC-Up	MF	GO:0032454	histone H3K9 demethylase activity	1/46	14/18496	0.034273	0.230192	0.195652	1
PBMC-Up	MF	GO:0032182	ubiquitin-like protein binding	2/46	119/18496	0.035346	0.230192	0.195652	2
PBMC-Up	MF	GO:0005523	tropomyosin binding	1/46	15/18496	0.036676	0.230192	0.195652	1
PBMC-Up	MF	GO:0016004	phospholipase activator activity	1/46	15/18496	0.036676	0.230192	0.195652	1
PBMC-Up	MF	GO:0031005	filamin binding	1/46	15/18496	0.036676	0.230192	0.195652	1
PBMC-Up	MF	GO:0086008	voltage-gated potassium channel activity involved in cardiac muscle cell action potential repolarization	1/46	15/18496	0.036676	0.230192	0.195652	1

PBMC-Up	MF	GO:1990782	protein tyrosine kinase binding	2/46	123/18496	0.037538	0.230192	0.195652	2
PBMC-Up	MF	GO:0015116	sulfate transmembrane transporter activity	1/46	16/18496	0.039074	0.230192	0.195652	1
PBMC-Up	MF	GO:0022858	alanine transmembrane transporter activity	1/46	16/18496	0.039074	0.230192	0.195652	1
PBMC-Up	MF	GO:0008242	omega peptidase activity	1/46	17/18496	0.041466	0.230192	0.195652	1
PBMC-Up	MF	GO:0140101	catalytic activity, acting on a tRNA	2/46	131/18496	0.042074	0.230192	0.195652	2
PBMC-Up	MF	GO:0045236	CXCR chemokine receptor binding	1/46	18/18496	0.043852	0.230192	0.195652	1
PBMC-Up	MF	GO:0060229	lipase activator activity	1/46	19/18496	0.046233	0.230192	0.195652	1
PBMC-Up	MF	GO:0070003	threonine-type peptidase activity	1/46	20/18496	0.048607	0.230192	0.195652	1

PBMC-		GO:003			22/18	0.053	0.230	0.195		
Up	MF	3038	bitter taste receptor activity	1/46	496	339	192	652	1	
PBMC-		GO:003			22/18	0.053	0.230	0.195		
Up	MF	3691	sialic acid binding	1/46	496	339	192	652	1	
PBMC-		GO:006			349/1	0.055	0.230	0.195		
Up	MF	1630	ubiquitin protein ligase activity	3/46	8496	749	192	652	3	
PBMC-		GO:001			24/18	0.058	0.230	0.195		
Up	MF	5295	solute:proton symporter activity	1/46	496	047	192	652	1	
PBMC-		GO:003			24/18	0.058	0.230	0.195		
Up	MF	5035	histone acetyltransferase binding	1/46	496	047	192	652	1	
PBMC-		GO:000			26/18	0.062	0.230	0.195		
Up	MF	1637	G protein-coupled chemoattractant receptor activity	1/46	496	733	192	652	1	
PBMC-		GO:000			26/18	0.062	0.230	0.195		
Up	MF	4950	chemokine receptor activity	1/46	496	733	192	652	1	
PBMC-		GO:009			26/18	0.062	0.230	0.195		
Up	MF	7602	cullin family protein binding	1/46	496	733	192	652	1	

PBMC-		GO:006			367/1	0.062	0.230	0.195	
Up	MF	1659	ubiquitin-like protein ligase activity	3/46	8496	909	192	652	3
PBMC-		GO:000			27/18	0.065	0.230	0.195	
Up	MF	5242	inward rectifier potassium channel activity	1/46	496	067	192	652	1
PBMC-		GO:001			27/18	0.065	0.230	0.195	
Up	MF	6866	intramolecular transferase activity	1/46	496	067	192	652	1
PBMC-		GO:000			28/18	0.067	0.230	0.195	
Up	MF	5452	solute:inorganic anion antiporter activity	1/46	496	396	192	652	1
PBMC-		GO:000			28/18	0.067	0.230	0.195	
Up	MF	8527	taste receptor activity	1/46	496	396	192	652	1
PBMC-		GO:014			28/18	0.067	0.230	0.195	
Up	MF	1052	histone H3 demethylase activity	1/46	496	396	192	652	1
PBMC-		GO:000			29/18	0.069	0.230	0.195	
Up	MF	5416	amino acid:monoatomic cation symporter activity	1/46	496	719	192	652	1
PBMC-		GO:003			29/18	0.069	0.230	0.195	
Up	MF	3612	receptor serine/threonine kinase binding	1/46	496	719	192	652	1

PBMC-		GO:001			30/18	0.072	0.230	0.195	
Up	MF	5106	bicarbonate transmembrane transporter activity	1/46	496	036	192	652	1
PBMC-		GO:003			30/18	0.072	0.230	0.195	
Up	MF	2452	histone demethylase activity	1/46	496	036	192	652	1
PBMC-		GO:014			30/18	0.072	0.230	0.195	
Up	MF	0457	protein demethylase activity	1/46	496	036	192	652	1
PBMC-		GO:000			31/18	0.074	0.230	0.195	
Up	MF	5310	dicarboxylic acid transmembrane transporter activity	1/46	496	348	192	652	1
PBMC-		GO:003			31/18	0.074	0.230	0.195	
Up	MF	1624	ubiquitin conjugating enzyme binding	1/46	496	348	192	652	1
PBMC-		GO:000		58/38	156/1	3.56E	1.04E	1.02E	
Down	BP	2181	cytoplasmic translation	1	8870	-58	-54	-54	58
PBMC-		GO:004		41/38	325/1	4.70E	6.90E	6.77E	
Down	BP	2254	ribosome biogenesis	1	8870	-21	-18	-18	41
PBMC-		GO:004		18/38	76/18	8.98E	8.78E	8.61E	
Down	BP	2273	ribosomal large subunit biogenesis	1	870	-15	-12	-12	18

PBMC-		GO:004		20/38	104/1	2.10E	1.54E	1.51E	
Down	BP	2274	ribosomal small subunit biogenesis	1	8870	-14	-11	-11	20
PBMC-		GO:007		26/38	243/1	4.47E	2.44E	2.40E	
Down	BP	1826	protein-RNA complex organization	1	8870	-12	-09	-09	26
PBMC-		GO:001		27/38	264/1	5.00E	2.44E	2.40E	
Down	BP	6072	rRNA metabolic process	1	8870	-12	-09	-09	27
PBMC-		GO:002		25/38	235/1	1.32E	5.55E	5.44E	
Down	BP	2618	protein-RNA complex assembly	1	8870	-11	-09	-09	25
PBMC-		GO:004		14/38	64/18	2.83E	1.04E	1.02E	
Down	BP	2255	ribosome assembly	1	870	-11	-08	-08	14
PBMC-		GO:000		24/38	225/1	3.24E	1.06E	1.04E	
Down	BP	6364	rRNA processing	1	8870	-11	-08	-08	24
PBMC-		GO:000			26/18	2.92E	8.56E	8.39E	
Down	BP	0027	ribosomal large subunit assembly	8/381	870	-08	-06	-06	8
PBMC-		GO:003		28/38	439/1	9.10E	2.43E	2.38E	
Down	BP	4470	ncRNA processing	1	8870	-08	-05	-05	28

PBMC-		GO:000			19/18	1.41E	0.000	0.000	
Down	BP	0028	ribosomal small subunit assembly	6/381	870	-06	346	339	6
PBMC-		GO:190			30/18	2.57E	0.005	0.005	
Down	BP	1798	positive regulation of signal transduction by p53 class mediator	6/381	870	-05	787	678	6
PBMC-		GO:014		22/38	408/1	3.14E	0.006	0.006	
Down	BP	0694	non-membrane-bounded organelle assembly	1	8870	-05	247	13	22
PBMC-		GO:000		11/38	120/1	3.20E	0.006	0.006	
Down	BP	7006	mitochondrial membrane organization	1	8870	-05	247	13	11
PBMC-		GO:000		19/38	336/1	5.78E	0.010	0.010	
Down	BP	0375	RNA splicing, via transesterification reactions	1	8870	-05	591	391	19
PBMC-		GO:000	RNA splicing, via transesterification reactions with bulged	18/38	332/1	0.000	0.024	0.024	
Down	BP	0377	adenosine as nucleophile	1	8870	153	903	435	18
PBMC-		GO:000		18/38	332/1	0.000	0.024	0.024	
Down	BP	0398	mRNA splicing, via spliceosome	1	8870	153	903	435	18
PBMC-		GO:003		23/38	496/1	0.000	0.031	0.030	
Down	BP	4655	nucleobase-containing compound catabolic process	1	8870	203	311	722	23

PBMC-Down	BP	GO:0000956	nuclear-transcribed mRNA catabolic process	10/381	128/18870	0.000274	0.040232	0.039475	10
PBMC-Down	BP	GO:0008380	RNA splicing	22/381	478/18870	0.000308	0.042518	0.041718	22
PBMC-Down	BP	GO:1901796	regulation of signal transduction by p53 class mediator	9/381	107/18870	0.000319	0.042518	0.041718	9
PBMC-Down	BP	GO:0006839	mitochondrial transport	12/381	190/18870	0.0005	0.06373	0.062531	12
PBMC-Down	BP	GO:1902253	regulation of intrinsic apoptotic signaling pathway by p53 class mediator	5/381	35/18870	0.000644	0.078728	0.077247	5
PBMC-Down	BP	GO:1901873	regulation of post-translational protein modification	15/381	284/18870	0.000699	0.081948	0.080406	15
PBMC-Down	BP	GO:0031125	rRNA 3'-end processing	3/381	10/18870	0.000882	0.099445	0.097574	3
PBMC-Down	BP	GO:0051438	regulation of ubiquitin-protein transferase activity	5/381	39/18870	0.00107	0.116187	0.114001	5

PBMC-Down	BP	GO:0009219	pyrimidine deoxyribonucleotide metabolic process	4/381	24/18870	0.001262	0.131424	0.128952	4
PBMC-Down	BP	GO:0006401	RNA catabolic process	17/381	367/18870	0.001358	0.131424	0.128952	17
PBMC-Down	BP	GO:1903320	regulation of protein modification by small protein conjugation or removal	13/381	243/18870	0.001389	0.131424	0.128952	13
PBMC-Down	BP	GO:0046902	regulation of mitochondrial membrane permeability	6/381	61/18870	0.001419	0.131424	0.128952	6
PBMC-Down	BP	GO:0009147	pyrimidine nucleoside triphosphate metabolic process	4/381	25/18870	0.001479	0.131424	0.128952	4
PBMC-Down	BP	GO:0009176	pyrimidine deoxyribonucleoside monophosphate metabolic process	3/381	12/18870	0.001569	0.131424	0.128952	3
PBMC-Down	BP	GO:0036444	calcium import into the mitochondrion	3/381	12/18870	0.001569	0.131424	0.128952	3
PBMC-Down	BP	GO:0071236	cellular response to antibiotic	3/381	12/18870	0.001569	0.131424	0.128952	3

PBMC-Down	BP	GO:1901028	regulation of mitochondrial outer membrane permeabilization involved in apoptotic signaling pathway	4/381	26/18870	0.00172	0.140112	0.137476	4
PBMC-Down	BP	GO:2001244	positive regulation of intrinsic apoptotic signaling pathway	6/381	64/18870	0.001821	0.144303	0.141588	6
PBMC-Down	BP	GO:1902369	negative regulation of RNA catabolic process	7/381	87/18870	0.001894	0.14613	0.143381	7
PBMC-Down	BP	GO:2000622	regulation of nuclear-transcribed mRNA catabolic process, nonsense-mediated decay	3/381	13/18870	0.002009	0.151045	0.148204	3
PBMC-Down	BP	GO:0000470	maturation of LSU-rRNA	4/381	28/18870	0.002282	0.167306	0.164159	4
PBMC-Down	CC	GO:0022626	cytosolic ribosome	55/402	118/19886	9.07E-62	3.84E-59	3.42E-59	55
PBMC-Down	CC	GO:0044391	ribosomal subunit	63/402	188/19886	1.01E-59	2.15E-57	1.91E-57	63
PBMC-Down	CC	GO:0005840	ribosome	64/402	246/19886	9.23E-53	1.30E-50	1.16E-50	64

PBMC-Down	CC	GO:0022625	cytosolic large ribosomal subunit	34/402	60/19886	2.64E-42	2.79E-40	2.48E-40	34
PBMC-Down	CC	GO:0015934	large ribosomal subunit	39/402	117/19886	5.43E-37	4.61E-35	4.09E-35	39
PBMC-Down	CC	GO:0022627	cytosolic small ribosomal subunit	22/402	41/19886	5.15E-27	3.64E-25	3.24E-25	22
PBMC-Down	CC	GO:0015935	small ribosomal subunit	25/402	75/19886	4.35E-24	2.63E-22	2.34E-22	25
PBMC-Down	CC	GO:0042788	polysomal ribosome	15/402	31/19886	6.65E-18	3.52E-16	3.13E-16	15
PBMC-Down	CC	GO:0005844	polysome	19/402	67/19886	4.18E-17	1.97E-15	1.75E-15	19
PBMC-Down	CC	GO:0032040	small-subunit processome	16/402	74/19886	1.33E-12	5.66E-11	5.03E-11	16
PBMC-Down	CC	GO:0030684	preribosome	17/402	109/19886	6.71E-11	2.59E-09	2.30E-09	17

PBMC-Down	CC	GO:0005925	focal adhesion	26/402	421/19886	5.15E-07	1.82E-05	1.62E-05	26
PBMC-Down	CC	GO:0030055	cell-substrate junction	26/402	431/19886	8.03E-07	2.62E-05	2.33E-05	26
PBMC-Down	CC	GO:0005791	rough endoplasmic reticulum	9/402	56/19886	1.69E-06	5.11E-05	4.54E-05	9
PBMC-Down	CC	GO:0098798	mitochondrial protein-containing complex	20/402	300/19886	3.45E-06	9.57E-05	8.51E-05	20
PBMC-Down	CC	GO:0005743	mitochondrial inner membrane	27/402	497/19886	3.61E-06	9.57E-05	8.51E-05	27
PBMC-Down	CC	GO:0097525	spliceosomal snRNP complex	10/402	96/19886	2.45E-05	0.000603	0.000536	10
PBMC-Down	CC	GO:0120114	Sm-like protein family complex	11/402	117/19886	2.56E-05	0.000603	0.000536	11
PBMC-Down	CC	GO:0030532	small nuclear ribonucleoprotein complex	10/402	106/19886	5.78E-05	0.00129	0.001147	10

PBMC-		GO:000		11/40	150/1	0.000	0.004	0.004	
Down	CC	1650	fibrillar center	2	9886	243	986	431	11
PBMC-		GO:009			16/19	0.000	0.004	0.004	
Down	CC	8554	cytoplasmic side of endoplasmic reticulum membrane	4/402	886	247	986	431	4
PBMC-		GO:004			45/19	0.000	0.005	0.004	
Down	CC	6540	U4/U6 x U5 tri-snRNP complex	6/402	886	275	182	606	6
PBMC-		GO:000		13/40	205/1	0.000	0.005	0.004	
Down	CC	5681	spliceosomal complex	2	9886	288	182	606	13
PBMC-		GO:009			46/19	0.000	0.005	0.004	
Down	CC	7526	spliceosomal tri-snRNP complex	6/402	886	311	182	606	6
PBMC-		GO:007			17/19	0.000	0.005	0.004	
Down	CC	1004	U2-type prespliceosome	4/402	886	318	182	606	4
PBMC-		GO:007			17/19	0.000	0.005	0.004	
Down	CC	1010	prespliceosome	4/402	886	318	182	606	4
PBMC-		GO:000			89/19	0.000	0.006	0.005	
Down	CC	0313	organellar ribosome	8/402	886	441	675	933	8

PBMC-Down	CC	GO:0005761	mitochondrial ribosome	8/402	89/19886	0.000441	0.006675	0.005933	8
PBMC-Down	CC	GO:0005684	U2-type spliceosomal complex	8/402	92/19886	0.000551	0.008061	0.007164	8
PBMC-Down	CC	GO:0030867	rough endoplasmic reticulum membrane	4/402	24/19886	0.001269	0.017931	0.015937	4
PBMC-Down	CC	GO:0106068	SUMO ligase complex	3/402	13/19886	0.002017	0.027584	0.024516	3
PBMC-Down	CC	GO:0016607	nuclear speck	18/402	417/19886	0.002205	0.029219	0.025969	18
PBMC-Down	CC	GO:0000930	gamma-tubulin complex	3/402	16/19886	0.003775	0.048505	0.04311	3
PBMC-Down	CC	GO:0098800	inner mitochondrial membrane protein complex	9/402	158/19886	0.004929	0.060127	0.053439	9
PBMC-Down	CC	GO:0005759	mitochondrial matrix	19/402	487/19886	0.005075	0.060127	0.053439	19

PBMC-Down	CC	GO:0014069	postsynaptic density	14/402	315/19886	0.005105	0.060127	0.053439	14
PBMC-Down	CC	GO:0000315	organellar large ribosomal subunit	5/402	56/19886	0.005402	0.060272	0.053569	5
PBMC-Down	CC	GO:0005762	mitochondrial large ribosomal subunit	5/402	56/19886	0.005402	0.060272	0.053569	5
PBMC-Down	CC	GO:0042575	DNA polymerase complex	3/402	19/19886	0.006246	0.067904	0.060352	3
PBMC-Down	CC	GO:0032279	asymmetric synapse	14/402	331/19886	0.007774	0.08241	0.073244	14
PBMC-Down	CC	GO:1904949	ATPase complex	8/402	146/19886	0.009785	0.101193	0.089938	8
PBMC-Down	CC	GO:0070603	SWI/SNF superfamily-type complex	6/402	92/19886	0.01091	0.110142	0.097892	6
PBMC-Down	CC	GO:0099572	postsynaptic specialization	14/402	346/19886	0.011187	0.110304	0.098036	14

PBMC-Down	CC	GO:0000793	condensed chromosome	12/402	281/19886	0.012292	0.118449	0.105275	12
PBMC-Down	CC	GO:1904813	ficolin-1-rich granule lumen	7/402	124/19886	0.013097	0.1234	0.109675	7
PBMC-Down	CC	GO:0098984	neuron to neuron synapse	14/402	362/19886	0.016012	0.147591	0.131176	14
PBMC-Down	CC	GO:0005688	U6 snRNP	2/402	11/19886	0.019872	0.175536	0.156013	2
PBMC-Down	CC	GO:0035145	exon-exon junction complex	2/402	11/19886	0.019872	0.175536	0.156013	2
PBMC-Down	CC	GO:0005758	mitochondrial intermembrane space	5/402	79/19886	0.021928	0.186497	0.165754	5
PBMC-Down	CC	GO:0035770	ribonucleoprotein granule	11/402	270/19886	0.021993	0.186497	0.165754	11
PBMC-Down	CC	GO:0042405	nuclear inclusion body	2/402	12/19886	0.023531	0.195634	0.173875	2

PBMC-Down	CC	GO:0005876	spindle microtubule	5/402	81/19886	0.024136	0.196805	0.174916	5
PBMC-Down	CC	GO:0000314	organellar small ribosomal subunit	3/402	32/19886	0.026367	0.207032	0.184005	3
PBMC-Down	CC	GO:0005763	mitochondrial small ribosomal subunit	3/402	32/19886	0.026367	0.207032	0.184005	3
PBMC-Down	CC	GO:0043240	Fanconi anaemia nuclear complex	2/402	13/19886	0.027443	0.211562	0.188032	2
PBMC-Down	CC	GO:0005685	U1 snRNP	3/402	33/19886	0.02858	0.216395	0.192327	3
PBMC-Down	MF	GO:0003735	structural constituent of ribosome	61/405	176/18496	8.60E-57	4.35E-54	4.21E-54	61
PBMC-Down	MF	GO:0019843	rRNA binding	11/405	66/18496	1.77E-07	4.47E-05	4.32E-05	11
PBMC-Down	MF	GO:0055105	ubiquitin-protein transferase inhibitor activity	5/405	11/18496	2.04E-06	0.000343	0.000332	5

PBMC-		GO:005			27/18	2.13E	0.002	0.002	
Down	MF	5106	ubiquitin-protein transferase regulator activity	6/405	496	-05	693	605	6
PBMC-		GO:004			25/18	0.000	0.018	0.017	
Down	MF	8027	mRNA 5'-UTR binding	5/405	496	182	391	791	5
PBMC-		GO:009			11/18	0.001	0.127	0.123	
Down	MF	7371	MDM2/MDM4 family protein binding	3/405	496	509	223	068	3
PBMC-		GO:003			13/18	0.002	0.149	0.144	
Down	MF	1386	protein tag activity	3/405	496	531	035	167	3
PBMC-		GO:014			13/18	0.002	0.149	0.144	
Down	MF	1047	molecular tag activity	3/405	496	531	035	167	3
PBMC-		GO:005			133/1	0.002	0.149	0.144	
Down	MF	1087	protein-folding chaperone binding	9/405	8496	651	035	167	9
PBMC-		GO:004		16/40	334/1	0.002	0.150	0.145	
Down	MF	5296	cadherin binding	5	8496	975	542	625	16
Nasal-		GO:003			83/18	4.52E	0.033	0.029	
Up	BP	1424	keratinization	4/45	870	-05	351	303	4

Nasal-		GO:004			317/1	0.000	0.037	0.032	
Up	BP	3588	skin development	6/45	8870	101	109	605	6
Nasal-		GO:000			245/1	0.000	0.053	0.046	
Up	BP	9913	epidermal cell differentiation	5/45	8870	283	215	756	5
Nasal-		GO:000			385/1	0.000	0.053	0.046	
Up	BP	8544	epidermis development	6/45	8870	288	215	756	6
Nasal-		GO:190	positive regulation of extrinsic apoptotic signaling pathway via		14/18	0.000	0.073	0.064	
Up	BP	2043	death domain receptors	2/25	870	497	342	44	2
Nasal-		GO:003			177/1	0.000	0.101	0.089	
Up	BP	0216	keratinocyte differentiation	4/45	8870	826	58	25	4
Nasal-		GO:000			83/18	0.001	0.107	0.094	
Up	BP	1895	retina homeostasis	3/45	870	019	443	401	3
Nasal-		GO:200			24/18	0.001	0.136	0.120	
Up	BP	0209	regulation of anoikis	2/25	870	484	935	314	2
Nasal-		GO:004			28/18	0.002	0.165	0.145	
Up	BP	4342	type B pancreatic cell proliferation	2/25	870	021	697	584	2

Nasal-Up	BP	GO:0043276	anoikis	2/25	34/18870	0.002972	0.211475	0.185806	2
Nasal-Up	BP	GO:0048730	epidermis morphogenesis	2/25	37/18870	0.003512	0.211475	0.185806	2
Nasal-Up	BP	GO:0001704	formation of primary germ layer	3/35	128/18870	0.003514	0.211475	0.185806	3
Nasal-Up	BP	GO:0001580	detection of chemical stimulus involved in sensory perception of bitter taste	2/25	39/18870	0.003896	0.211475	0.185806	2
Nasal-Up	BP	GO:1904994	regulation of leukocyte adhesion to vascular endothelial cell	2/25	41/18870	0.004298	0.211475	0.185806	2
Nasal-Up	BP	GO:0043542	endothelial cell migration	4/45	284/18870	0.004609	0.211475	0.185806	4
Nasal-Up	BP	GO:0016266	O-glycan processing	2/25	43/18870	0.004719	0.211475	0.185806	2
Nasal-Up	BP	GO:0050913	sensory perception of bitter taste	2/25	44/18870	0.004936	0.211475	0.185806	2

Nasal-		GO:005	detection of chemical stimulus involved in sensory perception of		45/18	0.005	0.211	0.185	
Up	BP	0912	taste	2/25	870	158	475	806	2
Nasal-		GO:001			47/18	0.005	0.218	0.191	
Up	BP	0830	regulation of myotube differentiation	2/25	870	615	101	627	2
Nasal-		GO:000			60/19	0.000	0.037	0.031	
Up	CC	1533	cornified envelope	3/46	886	361	588	197	3
Nasal-		GO:003			33/19	0.002	0.137	0.113	
Up	CC	1528	microvillus membrane	2/46	886	641	31	962	2
Nasal-		GO:004			469/1	0.004	0.153	0.127	
Up	CC	5177	apical part of cell	5/46	9886	418	154	111	5
Nasal-		GO:190			55/19	0.007	0.186	0.155	
Up	CC	4724	tertiary granule lumen	2/46	886	191	959	169	2
Nasal-		GO:003			62/19	0.009	0.188	0.156	
Up	CC	5580	specific granule lumen	2/46	886	063	518	462	2
Nasal-		GO:009			227/1	0.015	0.221	0.183	
Up	CC	8858	actin-based cell projection	3/46	9886	537	259	636	3

Nasal-Up	CC	GO:0005902	microvillus	2/46	97/19886	0.021205	0.221259	0.183636	2
Nasal-Up	CC	GO:0045095	keratin filament	2/46	97/19886	0.021205	0.221259	0.183636	2
Nasal-Up	CC	GO:0000815	ESCRT III complex	1/46	11/19886	0.025159	0.221259	0.183636	1
Nasal-Up	CC	GO:0043220	Schmidt-Lanterman incisure	1/46	11/19886	0.025159	0.221259	0.183636	1
Nasal-Up	CC	GO:1904930	amphisome membrane	1/46	12/19886	0.027415	0.221259	0.183636	1
Nasal-Up	CC	GO:0099523	presynaptic cytosol	1/46	13/19886	0.029666	0.221259	0.183636	1
Nasal-Up	CC	GO:0099524	postsynaptic cytosol	1/46	13/19886	0.029666	0.221259	0.183636	1
Nasal-Up	CC	GO:0043218	compact myelin	1/46	14/19886	0.031912	0.221259	0.183636	1

Nasal-		GO:004			14/19	0.031	0.221	0.183	
Up	CC	4753	amphisome	1/46	886	912	259	636	1
Nasal-		GO:003			322/1	0.038	0.223	0.185	
Up	CC	4774	secretory granule lumen	3/46	9886	28	577	56	3
Nasal-		GO:006			325/1	0.039	0.223	0.185	
Up	CC	0205	cytoplasmic vesicle lumen	3/46	9886	177	577	56	3
Nasal-		GO:003			326/1	0.039	0.223	0.185	
Up	CC	1983	vesicle lumen	3/46	9886	478	577	56	3
Nasal-		GO:004			18/19	0.040	0.223	0.185	
Up	CC	3034	costamere	1/46	886	846	577	56	1
Nasal-		GO:004			19/19	0.043	0.223	0.185	
Up	CC	4305	calyx of Held	1/46	886	067	946	866	1
Nasal-		GO:009			20/19	0.045	0.224	0.186	
Up	CC	9522	cytosolic region	1/46	886	282	255	122	1
Nasal-		GO:000			21/19	0.047	0.224	0.186	
Up	CC	5828	kinetochore microtubule	1/46	886	493	512	336	1

Nasal-		GO:004			160/1	0.052	0.239	0.198	
Up	CC	2581	specific granule	2/46	9886	879	107	449	2
Nasal-		GO:007			164/1	0.055	0.239	0.198	
Up	CC	0820	tertiary granule	2/46	9886	246	398	691	2
Nasal-		GO:000			10/18	0.000	0.014	0.010	
Up	MF	8330	protein tyrosine/threonine phosphatase activity	2/45	496	257	277	696	2
Nasal-		GO:003			10/18	0.000	0.014	0.010	
Up	MF	3550	MAP kinase tyrosine phosphatase activity	2/45	496	257	277	696	2
Nasal-		GO:001			13/18	0.000	0.016	0.012	
Up	MF	7017	MAP kinase tyrosine/serine/threonine phosphatase activity	2/45	496	444	422	303	2
Nasal-		GO:003			18/18	0.000	0.023	0.017	
Up	MF	3549	MAP kinase phosphatase activity	2/45	496	864	973	96	2
Nasal-		GO:000			96/18	0.001	0.036	0.027	
Up	MF	4725	protein tyrosine phosphatase activity	3/45	496	642	448	305	3
Nasal-		GO:003			36/18	0.003	0.064	0.047	
Up	MF	0280	structural constituent of skin epidermis	2/45	496	46	003	949	2

Nasal-<br>Up	MF	GO:014<br>0313	molecular sequestering activity	2/45	40/18<br>496	0.004<br>257	0.067<br>503	0.050<br>571	2
Nasal-<br>Up	MF	GO:000<br>8138	protein tyrosine/serine/threonine phosphatase activity	2/45	44/18<br>496	0.005<br>131	0.071<br>195	0.053<br>337	2
Nasal-<br>Up	MF	GO:001<br>9842	vitamin binding	3/45	150/1<br>8496	0.005<br>782	0.071<br>316	0.053<br>428	3
Nasal-<br>Up	MF	GO:000<br>4721	phosphoprotein phosphatase activity	3/45	176/1<br>8496	0.008<br>967	0.099<br>535	0.074<br>568	3
Nasal-<br>Up	MF	GO:001<br>7018	myosin phosphatase activity	2/45	82/18<br>496	0.016<br>992	0.139<br>071	0.104<br>188	2
Nasal-<br>Up	MF	GO:003<br>1419	cobalamin binding	1/45	10/18<br>496	0.024<br>071	0.139<br>071	0.104<br>188	1
Nasal-<br>Up	MF	GO:000<br>4722	protein serine/threonine phosphatase activity	2/45	100/1<br>8496	0.024<br>639	0.139<br>071	0.104<br>188	2
Nasal-<br>Up	MF	GO:000<br>5496	steroid binding	2/45	102/1<br>8496	0.025<br>561	0.139<br>071	0.104<br>188	2

Nasal-Up	MF	GO:0019864	IgG binding	1/45	11/18496	0.026446	0.139071	0.104188	1
Nasal-Up	MF	GO:0019911	structural constituent of myelin sheath	1/45	11/18496	0.026446	0.139071	0.104188	1
Nasal-Up	MF	GO:0001228	DNA-binding transcription activator activity, RNA polymerase II-specific	4/45	471/18496	0.027194	0.139071	0.104188	4
Nasal-Up	MF	GO:0016791	phosphatase activity	3/45	270/18496	0.027822	0.139071	0.104188	3
Nasal-Up	MF	GO:0001216	DNA-binding transcription activator activity	4/45	475/18496	0.027938	0.139071	0.104188	4
Nasal-Up	MF	GO:0004861	cyclin-dependent protein serine/threonine kinase inhibitor activity	1/45	12/18496	0.028816	0.139071	0.104188	1
Nasal-Up	MF	GO:0005527	macrolide binding	1/45	12/18496	0.028816	0.139071	0.104188	1
Nasal-Up	MF	GO:0008499	UDP-galactose:beta-N-acetylglucosamine beta-1,3-galactosyltransferase activity	1/45	12/18496	0.028816	0.139071	0.104188	1

Nasal-		GO:003			12/18	0.028	0.139	0.104	
Up	MF	5259	nuclear glucocorticoid receptor binding	1/45	496	816	071	188	1
Nasal-		GO:003			13/18	0.031	0.144	0.108	
Up	MF	1994	insulin-like growth factor I binding	1/45	496	181	211	039	1
Nasal-		GO:004			15/18	0.035	0.159	0.119	
Up	MF	8531	beta-1,3-galactosyltransferase activity	1/45	496	893	364	39	1
Nasal-		GO:007			16/18	0.038	0.160	0.120	
Up	MF	2542	protein phosphatase activator activity	1/45	496	24	879	526	1
Nasal-		GO:000			17/18	0.040	0.160	0.120	
Up	MF	5001	transmembrane receptor protein tyrosine phosphatase activity	1/45	496	582	879	526	1
Nasal-		GO:001			17/18	0.040	0.160	0.120	
Up	MF	9198	transmembrane receptor protein phosphatase activity	1/45	496	582	879	526	1
Nasal-		GO:000			19/18	0.045	0.162	0.121	
Up	MF	5520	insulin-like growth factor binding	1/45	496	249	022	382	1
Nasal-		GO:001			19/18	0.045	0.162	0.121	
Up	MF	9211	phosphatase activator activity	1/45	496	249	022	382	1

Nasal-Up	MF	GO:0030506	ankyrin binding	1/45	19/18496	0.045249	0.162022	0.121382	1
Nasal-Up	MF	GO:0008373	sialyltransferase activity	1/45	21/18496	0.049894	0.165975	0.124344	1
Nasal-Up	MF	GO:0046982	protein heterodimerization activity	3/45	344/18496	0.051	0.165975	0.124344	3
Nasal-Up	MF	GO:0140103	catalytic activity, acting on a glycoprotein	1/45	23/18496	0.054517	0.165975	0.124344	1
Nasal-Up	MF	GO:0042578	phosphoric ester hydrolase activity	3/45	358/18496	0.056186	0.165975	0.124344	3
Nasal-Up	MF	GO:0004806	triglyceride lipase activity	1/45	24/18496	0.05682	0.165975	0.124344	1
Nasal-Up	MF	GO:0016505	peptidase activator activity involved in apoptotic process	1/45	24/18496	0.05682	0.165975	0.124344	1
Nasal-Up	MF	GO:0019865	immunoglobulin binding	1/45	24/18496	0.05682	0.165975	0.124344	1

Nasal-Up	MF	GO:0004190	aspartic-type endopeptidase activity	1/45	26/18496	0.06141	0.168361	0.126131	1
Nasal-Up	MF	GO:0008320	protein transmembrane transporter activity	1/45	27/18496	0.063697	0.168361	0.126131	1
Nasal-Up	MF	GO:0070001	aspartic-type peptidase activity	1/45	27/18496	0.063697	0.168361	0.126131	1
Nasal-Up	MF	GO:0035250	UDP-galactosyltransferase activity	1/45	28/18496	0.065978	0.168361	0.126131	1
Nasal-Up	MF	GO:0043236	laminin binding	1/45	28/18496	0.065978	0.168361	0.126131	1
Nasal-Up	MF	GO:0001968	fibronectin binding	1/45	29/18496	0.068254	0.168361	0.126131	1
Nasal-Up	MF	GO:0051861	glycolipid binding	1/45	29/18496	0.068254	0.168361	0.126131	1
Nasal-Up	MF	GO:0008378	galactosyltransferase activity	1/45	31/18496	0.07279	0.173552	0.13002	1

Nasal-		GO:002			32/18	0.075	0.173	0.130	
Up	MF	2884	macromolecule transmembrane transporter activity	1/45	496	049	552	02	1
Nasal-		GO:007			32/18	0.075	0.173	0.130	
Up	MF	1889	14-3-3 protein binding	1/45	496	049	552	02	1
Nasal-		GO:003			37/18	0.086	0.195	0.146	
Up	MF	0291	protein serine/threonine kinase inhibitor activity	1/45	496	267	422	404	1
Nasal-		GO:000			39/18	0.090	0.197	0.147	
Up	MF	1530	lipopolysaccharide binding	1/45	496	717	443	918	1
Nasal-		GO:014			39/18	0.090	0.197	0.147	
Up	MF	0318	protein transporter activity	1/45	496	717	443	918	1
Nasal-		GO:000			224/1	0.103	0.217	0.163	
Up	MF	0287	magnesium ion binding	2/45	8496	073	684	083	2
Nasal-		GO:000			45/18	0.103	0.217	0.163	
Up	MF	8375	acetylglucosaminyltransferase activity	1/45	496	939	684	083	1
Nasal-		GO:014			477/1	0.109	0.224	0.167	
Up	MF	0297	DNA-binding transcription factor binding	3/45	8496	501	123	906	3

Nasal-Up	MF	GO:0005543	phospholipid binding	3/45	484/18496	0.113101	0.224123	0.167906	3
Nasal-Up	MF	GO:0016538	cyclin-dependent protein serine/threonine kinase regulator activity	1/45	50/18496	0.114814	0.224123	0.167906	1
Nasal-Up	MF	GO:0004879	nuclear receptor activity	1/45	52/18496	0.119128	0.224123	0.167906	1
Nasal-Up	MF	GO:0016504	peptidase activator activity	1/45	52/18496	0.119128	0.224123	0.167906	1
Nasal-Up	MF	GO:0098531	ligand-activated transcription factor activity	1/45	52/18496	0.119128	0.224123	0.167906	1
Nasal-Up	MF	GO:0050840	extracellular matrix binding	1/45	55/18496	0.12556	0.232287	0.174022	1
Nasal-Up	MF	GO:0016757	glycosyltransferase activity	2/45	268/18496	0.138289	0.251641	0.188522	2
Nasal-Up	MF	GO:0035091	phosphatidylinositol binding	2/45	276/18496	0.144956	0.259517	0.194423	2

Nasal-		GO:199			66/18	0.148	0.262	0.196	
Up	MF	0841	promoter-specific chromatin binding	1/45	496	754	091	351	1
Nasal-		GO:000		12/11	441/1	1.02E	0.009	0.009	
Down	BP	0280	nuclear division	0	8870	-05	926	66	12
Nasal-		GO:009		10/11	312/1	1.44E	0.009	0.009	
Down	BP	8813	nuclear chromosome segregation	0	8870	-05	926	66	10
Nasal-		GO:004		12/11	488/1	2.77E	0.012	0.012	
Down	BP	8285	organelle fission	0	8870	-05	717	376	12
Nasal-		GO:000		11/11	424/1	3.75E	0.012	0.012	
Down	BP	7059	chromosome segregation	0	8870	-05	935	588	11
Nasal-		GO:000			225/1	5.21E	0.014	0.013	
Down	BP	0819	sister chromatid segregation	8/110	8870	-05	347	962	8
Nasal-		GO:000			184/1	0.000	0.022	0.021	
Down	BP	0070	mitotic sister chromatid segregation	7/110	8870	102	351	752	7
Nasal-		GO:000			130/1	0.000	0.022	0.021	
Down	BP	7052	mitotic spindle organization	6/110	8870	114	351	752	6

Nasal-Down	BP	GO:0140014	mitotic nuclear division	8/110	274/18870	0.000204	0.035077	0.034136	8
Nasal-Down	BP	GO:0000381	regulation of alternative mRNA splicing, via spliceosome	4/110	54/18870	0.000276	0.042291	0.041157	4
Nasal-Down	BP	GO:1902850	microtubule cytoskeleton organization involved in mitosis	6/110	163/18870	0.000387	0.053328	0.051898	6
Nasal-Down	BP	GO:0000380	alternative mRNA splicing, via spliceosome	4/110	71/18870	0.000787	0.098537	0.095895	4
Nasal-Down	BP	GO:0051983	regulation of chromosome segregation	5/110	131/18870	0.001021	0.117249	0.114106	5
Nasal-Down	BP	GO:0007051	spindle organization	6/110	202/18870	0.001189	0.126046	0.122666	6
Nasal-Down	BP	GO:0051593	response to folic acid	2/110	11/18870	0.00179	0.176143	0.17142	2
Nasal-Down	BP	GO:0000727	double-strand break repair via break-induced replication	2/110	12/18870	0.002139	0.19653	0.191261	2

Nasal-Down	CC	GO:0000793	condensed chromosome	7/119	281/19886	0.001522	0.126012	0.121474	7
Nasal-Down	CC	GO:0071162	CMG complex	2/119	11/19886	0.001885	0.126012	0.121474	2
Nasal-Down	CC	GO:0031261	DNA replication preinitiation complex	2/119	13/19886	0.002653	0.126012	0.121474	2
Nasal-Down	CC	GO:1990907	beta-catenin-TCF complex	2/119	13/19886	0.002653	0.126012	0.121474	2
Nasal-Down	CC	GO:0005819	spindle	8/119	431/19886	0.004417	0.145995	0.140737	8
Nasal-Down	CC	GO:0000775	chromosome, centromeric region	6/119	258/19886	0.00461	0.145995	0.140737	6

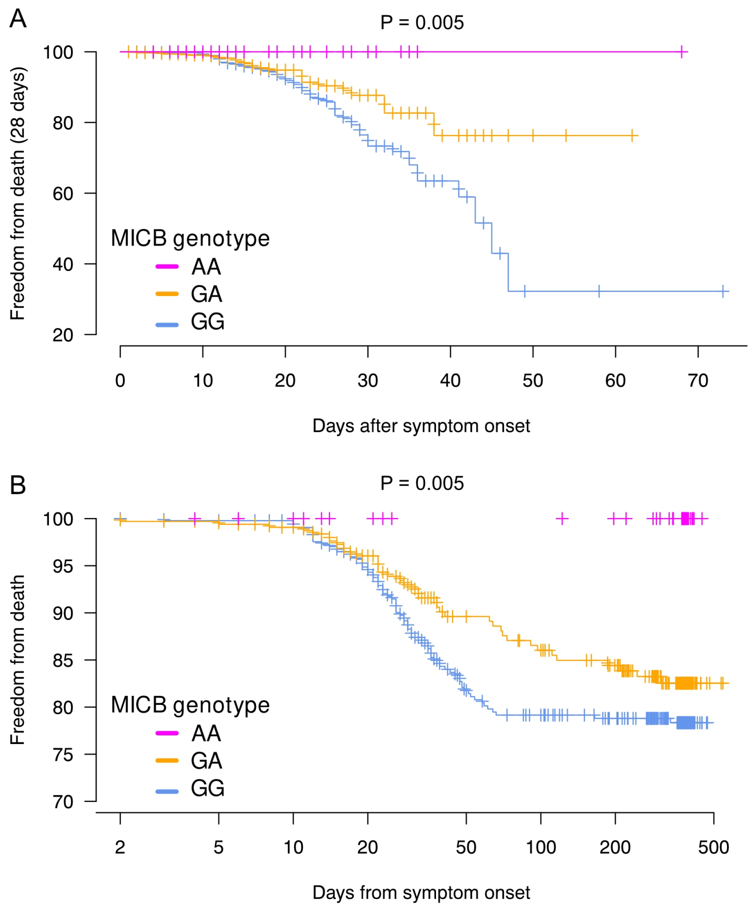

Supplemental Figure 1. COVID-19 mortality by MICB^{G406A}polymorphism

Survival analysis of death on days from symptom onset **A**) by 28-days post-admission or **B**) ever during the study and number of copies of the MICB^{G406A} variant allele were compared by Cox proportional-hazards models. Results for patients with no copies of the variant allele (GG) are shown in blue, one copy of the variant allele (GA) in orange) and two copies of the variant allele (AA) in magenta.

A

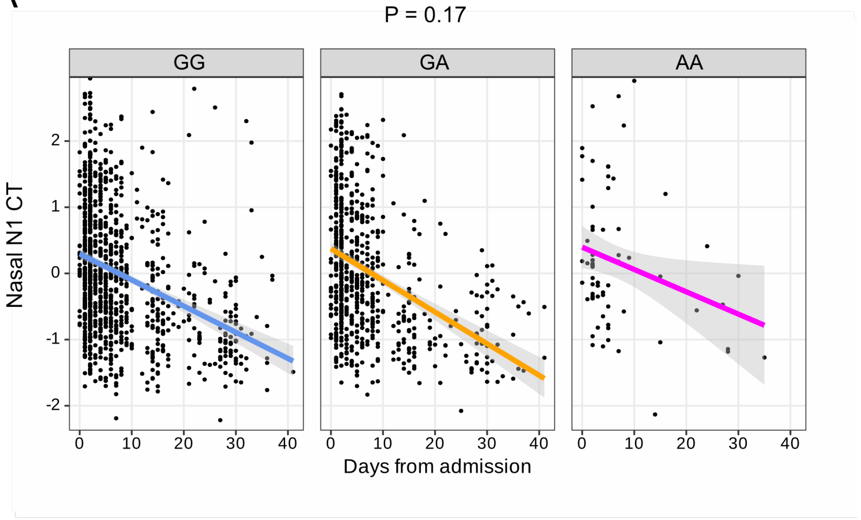

B

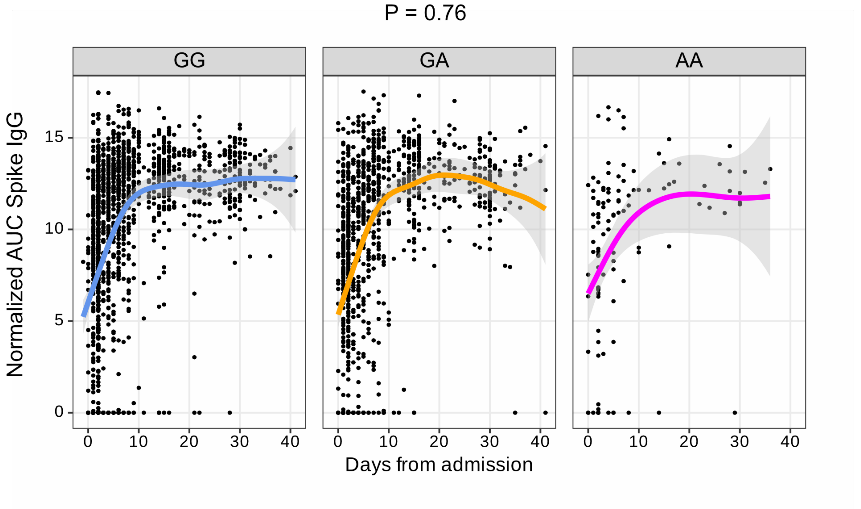

Supplemental Figure 2. Longitudinal nasal SARS-CoV-2 viral load and serum anti-Spike IgG by MICB^{G406A} polymorphism

Longitudinal **A**) nasal SARS-CoV-2 viral load (N1 CT value) and **B**) serum anti-spike IgG levels (AUC) by number of copies of the MICB^{G406A} variant allele were compared by linear regression, including patient as a random effect. Results for patients with no copies of the variant allele (GG) are shown in blue, one copy of the variant allele (GA) in orange) and two copies of the variant allele (AA) in magenta.

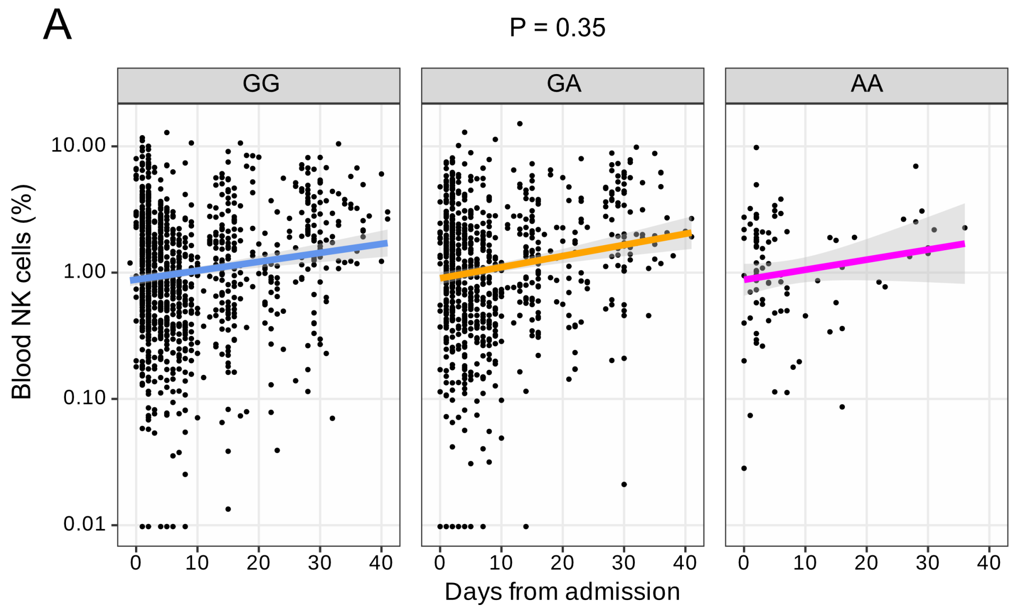

Supplemental Figure 3. Longotudinal NK cell frequency in PBMC by MICB^{G406A}polymorphism

Blood NK cell frequency **A**) longitudinally by number of copies of the MICB^{G406A} variant allele were compared by linear regression, including patient identifier as a random effect. Results for patients with no copies of the variant allele (GG) are shown in blue, one copy of the variant allele (GA) in orange) and two copies of the variant allele (AA) in magenta.

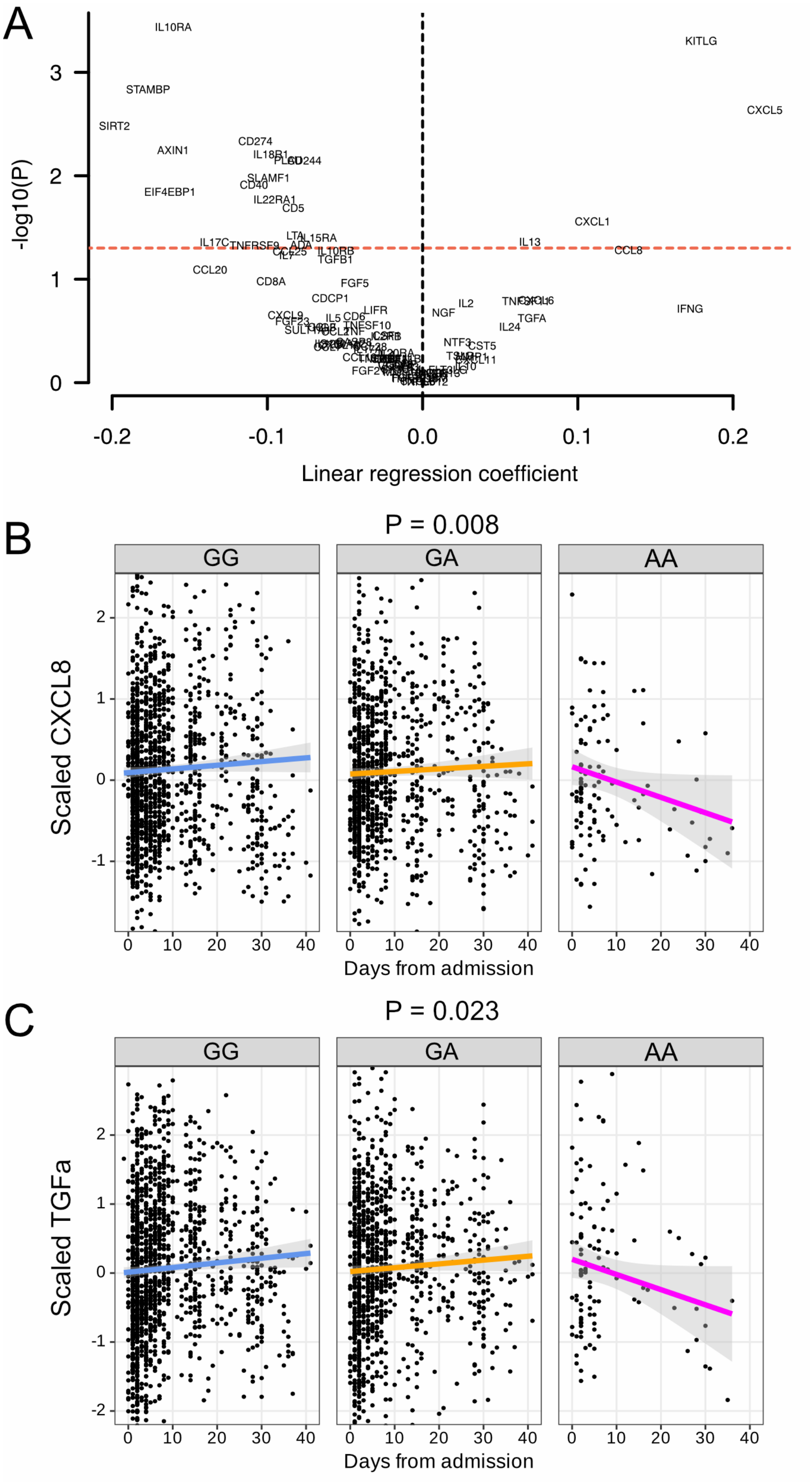

A
B
C
-log10(P)
Linear regression coefficient
IL10RA
KITLG
CXCL5
STAMBP
SIRT2
AXIN1
CD274
EIF4EBP1
CXCL1
IL13
CCL8
CCL20
IFNG
P = 0.008
P = 0.023
GG
GA
AA
Scaled CXCL8
Scaled TGFa
Days from admission

199

Supplemental Figure 4. Serum proteins by $MICB^{G406A}$polymorphism

Visit-1 serum protein levels by number of copies of the $MICB^{G406A}$ variant allele were compared by linear regression. **A**) Linear regression coefficient against -log10 transformed P-values. Longitudinal **B-C**) serum protein levels by number of copies of the $MICB^{G406A}$ variant allele were compared by linear regression, including patient as a random effect. Results for patients with no copies of the variant allele (GG) are shown in blue, one copy of the variant allele (GA) in orange) and two copies of the variant allele (AA) in magenta.

Conflict of interest: The Icahn School of Medicine at Mount Sinai has filed patent applications relating to SARS-CoV-2 serological assays, NDV-based SARS-CoV-2 vaccines influenza virus vaccines, and influenza virus therapeutics, which list Florian Krammer as coinventor. Mount Sinai has spun out a company, Kantaro, to market serological tests for SARS-CoV-2 and another company, Castlevax, to develop SARS-CoV-2 vaccines. FK is cofounder and scientific advisory board member of Castlevax. FK has consulted for Merck, Curevac, Seqirus, GSK, and Pfizer and is currently consulting for 3rd Rock Ventures, Sanofi, Gritstone, and Avimex. The Krammer laboratory is also collaborating with Dynavax on influenza vaccine development and with VIR on influenza virus therapeutics development. VS is a coinventor on a patent filed relating to SARS-CoV-2 serological assays (the “Serology Assays”). OL is a named inventor on patents held by Boston Children’s Hospital relating to vaccine adjuvants and human in vitro platforms that model vaccine action. His laboratory has received research support from GlaxoSmithKline (GSK) and is a cofounder of and advisor to Ovax Inc. C. Cairns serves as a consultant to bioMerieux and is funded for a grant from Bill & Melinda Gates Foundation. JAO is a consultant at Knocean Inc. Jessica Lasky-Su serves as a scientific advisor of Precion Inc. SRH, GM, and KW are employees of Metabolon Inc. VSM is a current employee of MyOwnMed. NR reports grants or contracts with Merck, Sanofi, Pfizer, Vaccine Company, and Immorna and has participated on data safety monitoring boards for Moderna, Sanofi, Seqirus, Pfizer, EMMES, ICON, BARDA, CyanVan, and Imunon Micron. NR has also received support for meetings/travel from Sanofi and Moderna and honoraria from Virology Education and Krog consulting. C. Cotsapas is a current employee of Vesalius Therapeutics. AR is a current employee of Immunai Inc. SK is a consultant related to ImmPort data repository for Peraton. NG is a consultant for Tempus Labs and the National Basketball Association. AI is a consultant for 4BIO, Blue Willow Biologics, Revelar Biotherapeutics, RIGImmune, Xanadu Bio, and Paratus Sciences. MK receives research funds paid to her institution from NIH. ALA is affiliated with Sanofi and Astra-Zeneca for work in asthma; serves as a consultant for Astra-Zeneca, Sanofi, Chiesi, GSK for severe asthma; and is a cofounder and CMO for RaeSedo Inc., a company created to develop peptidomimetics for treatment of inflammatory lung disease **(4. AUTHOR: Does edit for syntax retain meaning? If not, clarify.)**. EM received research funding from Babson Diagnostics and honorarium from Multiple Sclerosis Association of America and has served on the advisory boards of Genentech, Horizon, Teva, and Viela Bio. C. Calfee receives research funding from NIH, FDA, DOD, Roche-Genentech, and Quantum Leap Healthcare Collaborative as well as consulting services for Janssen, Vasomune, Gen1e Life Sciences, NGMBio, and Cellenkos. WS was an investigator for a research agreement, through Yale University, from the Shenzhen Center for Health Information for work to advance intelligent disease prevention and health promotion; collaborates with the National Center for Cardiovascular Diseases in Beijing; is a technical consultant to Hugo Health, a personal health information platform; nd is a cofounder of Refactor Health, an AI-augmented data management platform for health care; and has received grants from Merck and Regeneron Pharmaceutical for research related to COVID-19. GAM received research grants from Rehdhill, Cognivue, Pfizer, and Genentech and served as a research consultant for Gilead, Merck, Viiv/GSK, and Janssen. LNG received research funding paid to her institution from Pfizer Inc. CH receives support through her institution from the NIH and CDC.
